# Chemical Constituents With Antiproliferative Activity From *Pogostemon cablin* (Blanco) Benth.

**DOI:** 10.3389/fchem.2022.938851

**Published:** 2022-07-15

**Authors:** Xingjia Peng, Song Ang, Yizi Zhang, Fenling Fan, Mengshuo Wu, Peiting Liang, Yan Wen, Lishe Gan, Kun Zhang, Dongli Li, Jianmin Yue

**Affiliations:** ^1^ School of Biotechnology and Health Sciences, Wuyi University, Jiangmen, China; ^2^ International Healthcare Innovation Institute (Jiangmen), Jiangmen, China; ^3^ School of Chemical Engineering and Light Industry, Guangdong University of Technology, Guangzhou, China; ^4^ Key Laboratory of Drug Research, Shanghai Institute of Materia Medica, Chinese Academy of Sciences, Shanghai, China

**Keywords:** *Pogostemon cablin*, sesquiterpenoid, flavonoid, antiproliferative activity, apoptosis

## Abstract

Two new patchoulene sesquiterpenoid glycosides (**1–2**), a natural patchoulane-type sesquiterpenoid (**3**) and a natural cadinene-type sesquiterpenoid (**4**), were isolated from the aerial parts of *Pogostemon cablin* (Blanco) Benth., together with eleven known sesquiterpenoids (**5**–**15**) and eleven known flavonoids (**16**–**26**). Their chemical structures were elucidated on the basis of spectroscopic methods, including NMR, HRESIMS, IR, and CD spectroscopic data analysis, as well as chemical hydrolysis. The isolated compounds **1**–**13** and **15**–**26** were tested for inhibitory effects on the proliferation of HepG2 cancer cells. Among them, compounds **17** and **19** displayed anti-proliferative effects against HepG2 cells with IC_50_ values of 25.59 and 2.30 μM, respectively. Furthermore, the flow cytometry analysis and Western blotting assays revealed that compound **19** significantly induced apoptosis of HepG2 cells by downregulating the ratio of Bcl-2/Bax and upregulating the expression of cleaved caspase-3 and cleaved caspase-9. Therefore, the potential pharmaceutical applications of *P. cablin* would be applied according to our study findings.

## Introduction

*Pogostemon cablin* (Blanco) Benth. is an annual herb known as patchouli, a form of traditional Chinese medicine for the treatment of upset stomach, vomiting, diarrhea, headache, and fever. The plant, a member of the genus *Pogostemon*, Lamiaceae family, was native to South and Southeast Asia, such as Indonesia, Malaysia, Philippines, and India, and was introduced to China in the 9th century as a spice. It was fostered in Guangdong, Guangxi, Fujian, and Taiwan provinces in China (Feng et al., 1994). In recent years, a large number of phytochemical studies on *P. cablin* had been carried out to concentrate on the constituents of this plant, which showed the presence of various monoterpenes and sesquiterpenoids (Hikino et al., 1968; Terhune et al., 1973), triterpenoids (Huang et al., 2009), steroids (Kongkathip et al., 2009), flavonoids (Ding et al., 2009), alkaloids (Büchi et al., 1966), and glycosides (Wang et al., 2010). The reported constituents from *P. cablin* possessed marked activities such as antibacterial activity, anti-influenza virus, anti-inflammation, cytotoxicity, antimutagenic activity, antiplatelet aggregation, and insecticidal activity (Li et al., 2013). In our continuous phytochemical studies on the constituents of this medicinal herb, two new patchoulene sesquiterpenoid glycosides (**1–2**), a natural patchoulane-type sesquiterpenoid (**3**) and a natural cadinene-type sesquiterpenoid (**4**), as well as eleven known sesquiterpenoids (**5–15**) and eleven known flavonoids (**16–26**), were isolated and characterized. In addition, the anti-proliferative activities of the isolated compounds (**1**–**13** and **15**–**26**) against HepG2 cancer cells were assessed in this article. Furthermore, the apoptosis-inducing effects of compound **19** in HepG2 cells were also investigated in the current study. To the best of our knowledge, the apoptosis-induced activity of compound **19** is reported for the first time.

## Experiment

### General Experimental Procedures

Column chromatographies (CC) were carried out with silica gel (200–300 mesh, Qingdao Marine Chemical Factory), silica gel for chromatography C_18_ SMB 100–20/45 (Fuji Silysia Chemical Ltd), and Sephadex LH-20 (Pharmacia Biotech AB). TLC was performed using precoated silica gel GF_254_ plates (Yantai Chemical Industry Research Institute). MPLC (medium pressure liquid chromatography) was carried out on a Buchi Pure C-815 apparatus. High-performance liquid chromatography (HPLC) was carried out on a Waters 1500-Series system. The semi-preparative C_18_ column used was the SunFire^®^ C_18_ OBD (250 × 10 mm) apparatus. UPLC was carried out on a Waters Acquity UPLC-Class instrument. IR data were obtained with a KBr pellet on a Thermo Scientific Fourier Transform NICOLET iS5 Infrared Spectrometer (Waltham, USA). High-resolution electrospray ion mass (HRESIMS) was performed on a Thermo Scientific Q-Exactive mass spectrometer (Waltham, USA). 1D and 2D NMR data were recorded on a Bruker AVANCE NEO 500 spectrometer (Bremen, Germany) with chloroform-*d* and methanol-*d*_*4*_ as solvents. Chemical shift values were expressed in *δ* (ppm) relative to tetramethylsilane (TMS) as the internal standard. An Anton Paar MCP 200 automatic polarimeter (Graz, Austria) was used to determine optical rotations in ACN at 25°C. CD spectra were obtained on a Chirascan spectrometer (England, United Kingdom) at room temperature using a 0.2-cm standard cell. All solvents used in column chromatography and HPLC were of analytical grade (Guangzhou Chemical Reagents Company Ltd., Guangzhou, China) and chromatographic grade (Thermo Fisher), respectively.

### Plant Material

The aerial parts of *P*. *cablin* were collected in September 2019, GuangXi Province, China. The plant material was identified by Professor Xiaoji Zheng, and a voucher specimen (201909PC) was deposited at Wuyi University, Jiangmen, China.

### Extraction and Isolation

The air-dried aerial parts of *P. cablin* (13.0 kg) were powdered and extracted with 95% ethanol (3 × 30 L) at room temperature for 6 days. The extract was evaporated under reduced pressure (45°C) to afford a brown residue (2.0 kg), which was suspended in water (5 L) and extracted sequentially with petroleum ether, EtOAc (ethyl acetate), and *n*-BuOH at room temperature. The EtOAc fraction (178 g) was subjected to a silica gel (200–300 mesh) column using a gradient petroleum ether (PE)-EtOAc system (100:1, 50:1, 30:1, 10:1, 5:1, 1:1, and 0:1, each 15 L) as eluents to afford 19 fractions (Fr. 1–Fr. 19). Fr. 4 (2.8 g) was subjected to Sephadex LH-20 and yielded two subfractions, namely, (Fr. 4.1- Fr. 4.2) and Fr. 4.2 (1.2 g), which were further purified by using a reversed phase (C_18_ SMB 100–20/45, 30 g, H_2_O-MeOH: 40:1 to 0:100) column to obtain compound **10** (432 mg). Fr. 7 (4.5 g) was submitted to a silica gel (200–300 mesh) column using PE and EtOAc as eluents (50:1 to 0:1) to afford five subfractions (Fr. 7.1–Fr. 7.5); Fr. 7.4 (950 mg) was submitted to Sephadex LH-20 and eluted with the CHCl_2_–CH_3_OH (1:1) system to obtain compound **12** (7 mg), Fr. 7.3 (530 mg) was submitted to Sephadex LH-20 and eluted with the CHCl_2_–CH_3_OH (1:1) system to afford compounds **18** (77 mg) and **24** (44 mg). Fr. 8 (5.5 g) was subjected to Sephadex LH-20 and eluted with the CHCl_2_–CH_3_OH (1:1) system to afford two subfractions (Fr. 8.1 and Fr. 8.2), and Fr. 8.2 (1.6 g) was further purified by using a silica gel (200–300 mesh) column using PE and EtOAc as eluents (100:1 to 0:1) to obtain compound **23** (373 mg). Fr. 9 (15.5 g) was subjected to a reversed phase (C_18_ SMB 100–20/45, 30 g, H_2_O-MeOH: 30:1 to 0:100) column, and four subfractions (Fr. 9.1–Fr. 9.4) were yielded, and Fr. 9.1 (200 mg) was further purified by semi-preparative HPLC (0–60 min: isocratic 80% CH_3_CN in water) to yield compounds **4** (25 mg) and **5** (15 mg); Fr. 9.2 (330 mg) was purified by semi-preparative HPLC (0–60 min: isocratic 80% CH_3_CN in water) to obtain **6** (27 mg) and **9** (20 mg); Fr. 9.4 (500 mg) was subjected to semi-preparative HPLC (0–60 min: isocratic 80% CH_3_CN in water) to afford compounds **16** (4 mg), **20** (14 mg), and **25** (48 mg). Fr. 10 (4.1 g) was submitted to a silica gel (200–300 mesh) column using PE and EtOAc as eluents (30:1 to 0:1) to obtain Fr. 10.1–Fr. 10.3, and then, Fr. 10.3 (2.0 g) was further purified by Sephadex LH-20 to obtain compound **19** (75 mg). Fr. 12 (5.4 g) was subjected to Sephadex LH-20 to afford two subfractions (Fr. 12.1 and Fr. 12.2). Fr. 12.1 (1.3 g) was further purified using a reversed phase (C_18_ SMB 100–20/45, 30 g, H_2_O-MeOH: 30:1 to 0:100) column to obtain compounds **3** (20 mg) and **22** (5 mg). Fr. 12.2 (150 mg) was subjected to semi-preparative HPLC (0–60 min: isocratic 60% CH_3_CN in water) to afford compounds **7** (1 mg), **13** (1 mg), **14** (1 mg), and **17** (41 mg). Fr. 13 (1.3 g) was submitted to a silica gel (200–300 mesh) column using PE and EtOAc as eluents (50:1 to 0:1) to yield two subfractions (Fr. 13.1 and Fr. 13.2), and Fr. 13.1 (300 mg) was subjected to Sephadex LH-20 and a reversed phase (C_18_ SMB 100–20/45, 30 g, H_2_O-MeOH: 30:1 to 0:100) column to obtain compounds **11** (5 mg), **21** (8 mg), **8** (26 mg), and **26** (12 mg). Fr. 15 (6.3 g) was submitted to a silica gel (200–300 mesh) column using PE and EtOAc as eluents (30:1 to 0:1) and further purified by using a reversed phase (C_18_ SMB 100–20/45, 30 g, H_2_O-MeOH: 40:1 to 0:100) column to obtain compound **15** (5 mg). Fr. 19 (11.5 g) was subjected to a silica gel column (200–300 mesh, 5 × 40 cm, 120 g) using a gradient CH_2_Cl_2_-MeOH system (10:1, 5:1, 1:1, 0:1) to obtain two subfractions (Fr. 19.1 and Fr. 19.2). Fr. 19.1 (2.0 g) was submitted to a reversed phase (C_18_ SMB 100–20/45, 30 g, H_2_O-MeOH: 50:1 to 0:100) column and semi-preparative HPLC (0–60 min: isocratic 40% CH_3_CN in water) to yield compounds **1** (6 mg) and **2** (6 mg).

### Compound Characterization

Compound **1**: an amorphous white powder; [*α*]^25^ D = −35.5 (*c* 0.1, MeOH); IR (KBr) *ν*_max_ 3,449, 1,638, 1,075, and 536 cm^−1^; ^1^H and ^13^C NMR data, see Table 1 and Table 2; HRESIMS *m*/*z* 427.2318 [M + HCOO]^-^ (calcd for C_22_H_35_O_8_, 427.2326).

**TABLE 1:** ^1^H NMR data of compounds **1** and **2** in CD_3_OD and of compounds **3** and **4** in CDCl_3_ (500 MHz).

Position	1	2	3	4
1	—	—	—	—
2	5.02, m	2.25, m	1.77, dd (14.0, 5.7)	2.20, m
	—	—	1.51, m	1.38, d (2.6)
3	2.44, ddt (16.5, 7.9, 2.2)	1.99, m	1.59, dd (12.6, 5.7)	2.24, m
	2.33, dq (16.5, 2.4)	1.72, m	1.40, td (12.6, 5.8)	1.64, m
4	2.58, m	2.71, m	2.17, m	2.54, m
5	2.94, m	—	1.20, m	—
6	1.73, d (12.7)	1.93, m	1.85, m	3.01, d (12.1)
	1.42, ddd (12.7, 7.7, 3.6)	1.28, d (9.6)	1.25, m	—
7	1.78, m	1.76, m	1.64, m	2.38, td (12.1, 4.3)
8	1.96, m	2.36, m	1.48, dd (5.8, 3.0)	1.27, overlapped
	1.54, m	1.71, m	1.32, m	—
9	1.66, m	1.73, m	1.87, m	1.95, tt (13.7, 3.8)
	1.52, m	1.70, m	1.06, m	1.38, m
10	—	—	—	1.73, m
11	—	—	—	1.10, d (7.2)
12	0.80, s	0.91, s	1.07, s	—
13	0.90, s	0.91, s	1.07, s	4.65, m
	—	—	—	4.50, s
14	4.01, dd (9.0, 6.0)	3.97, dd (9.2, 4.9)	0.84, s	1.80, s
	3.26, d (6.0)	3.27, m	—	—
15	1.02, s	0.93, s	3.89, dd (14.0, 7.7)	1.20, d (7.3)
	—	—	3.87, dd (14.0, 7.0)	—
16	—	—	—	—
17	—	—	2.04, s	—
1′	4.22, d (7.9)	4.23, d (7.9)	—	—
2′	3.16, dd (9.0, 7.9)	3.16, dd (9.0, 7.9)	—	—
3′	3.27, dd (7.9, 4.0)	3.26, dd (7.9, 4.0)	—	—
4′	3.26, m	3.27, m	—	—
5′	3.35, m	3.34, m	—	—
6′	3.86, dd (11.8, 1.7)	3.86, dd (11.9, 1.8)	—	—
	3.66, dd (11.8, 5.2)	3.66, dd (11.9, 5.3)	—	—

**TABLE 2:** ^13^C NMR data of compounds **1** and **2** in CD_3_OD and of compounds **3** and **4** in CDCl_3_ (125 MHz).

Position	1	2	3	4
1	154.0	146.7	75.8	80.1
2	116.5	31.3	31.8	31.3
3	36.9	28.1	23.5	28.5
4	40.0	50.0	33.3	44.8
5	42.6	131.9	38.9	214.6
6	29.8	30.0	24.2	50.3
7	47.9	46.1	39.1	38.5
8	27.4	34.4	24.7	26.8
9	36.7	41.5	28.7	28.1
10	47.8	44.2	37.2	39.8
11	45.3	46.4	40.2	16.1
12	20.7	19.7	26.8	150.8
13	24.2	23.9	24.3	107.2
14	72.7	73.8	20.6	22.7
15	17.0	15.5	66.9	15.8
16	—	—	171.4	—
17	—	—	21.1	—
1′	104.8	104.7	—	—
2′	75.2	75.2	—	—
3′	77.9	77.9	—	—
4′	71.7	71.7	—	—
5′	78.2	78.2	—	—
6′	62.8	62.8	—	—

Compound **2**: an amorphous white powder; [α]^25^ D = −25.3 (*c* 0.1, MeOH); IR (KBr) *ν*_max_ 3,449, 2,947, 1,638, 1,384, 1,077, and 536 cm^−1^; ^1^H and ^13^C NMR data, see Table 1 and Table 2; HRESIMS *m*/*z* 427.2320 [M + HCOO]^-^ (calcd for C_22_H_35_O_8_, 427.2326).

Compound **3**: colorless oil; [*α*]^25^ D = −37.0 (*c* 0.1, MeOH); IR (KBr) *ν*_max_ 3,449, 2,964, 2,956, 2,922, 2,851, 1,685, 1,603, 1,261, 1,166, 1,105 and 1,026 cm^−1^; ^1^H and ^13^C NMR data, see Table 1 and Table 2; HRESIMS *m*/*z* 281.2109 [M + H]^+^ (calcd for C_17_H_29_O_3_, 281.2111).

Compound **4**: an amorphous white powder; [*α*]^25^ D = −38.0 (*c* 0.1, MeOH); IR (KBr) *ν*_max_ 3,474, 2,962, 2,923, 2,856, 1707, 1,644, 1,451, and 1,394 cm^−1^; ^1^H and ^13^C NMR data, see Table 1 and Table 2; HRESIMS *m*/*z* 237.1849 [M + H]^+^ (calcd for C_15_H_25_O_2_, 237.1849).

### Quantum Chemical ECD Calculation

Electronic circular dichroism (ECD) was applied to establish the absolute configurations of **1′** and **2′**, according to the reported method (Stephens and Harada, 2010). According to the key correlations observed in the NOESY spectrum, CHEM3D software with the MM2 force field was applied to search the preliminary conformational distribution. Geometric optimization of compounds **1′** and **2′** was calculated with the density functional theory (DFT) method and time-dependent DFT (TDDFT) *via* the Gaussian 09 program (Gaussian, Inc., Wallingford CT, USA). The optimized conformers obtained were submitted to CD calculation by the TDDFT [B3LYP/6-31G(d)] method. The computational data were fitted in the Origin 2021 (OriginLab Corporation, Northampton, MA, USA).

### Acid Hydrolysis

Each compound (1–2 mg) was hydrolyzed with 1 M HCl (4 mL) for 4 h at 80°C. The reaction mixture was extracted with ethyl acetate 3 times (Zhang et al., 2020). The water layer was evaporated repeatedly under reduced pressure with MeOH until dryness, giving a monosaccharide residue. It was dissolved in anhydrous pyridine (1 mL), and L-cysteine methyl ester hydrochloride (3 mg) was added. The mixture was kept at 60°C for 1 h. Then, 5 μL (0.5 mg) *O*-tolylisothiocyanate was added and stirred at 60°C for another 1 h. The reaction mixture was directly analyzed by reversed-phase HPLC. The absolute configurations of sugars of compounds **1** and **2** were determined by comparing the retention times with derivatives of standard sugars prepared in a similar manner. Retention times for derivatives were 12.2 min (L-glucose) and 12.9 min (D-glucose), respectively. Meanwhile, compounds **1** and **2** were hydrolyzed to obtain compounds **1′** and **2′**, respectively.

### Anti-Proliferative Activity Assay (MTT Assay)

The antiproliferative activity of the isolated compounds was evaluated against the growth of hepatocellular carcinoma cells HepG2 (cells were grown in Dulbecco’s modified Eagle medium containing 10% FBS at 37°C) using an MTT assay. HepG2 cells were seeded in 96-well plates at a density of 5,000 cells per well and cultured in a 37°C incubator for 24 h (Tang et al., 2020). After 24 h, the medium was removed, and a fresh medium containing different compounds with a series of concentration gradients was added to each well and cultured in a 37°C incubator for 72 h. A measure of 20 μL (5 mg/mL) of the MTT solution was added to each well and cultured in a 37°C incubator for 4 h. Then, the supernatant was removed, and DMSO (100 μL) was added to each well. The optical density was measured at a wavelength of 490 nm after 15 min of shaking. The IC_50_ values of each compound were calculated with GraphPad Prism 8.0 software (GraphPad Software Inc., San Diego, CA, USA).

### Flow Cytometry

HepG2 cells were seeded in 6-well plates (1 × 10^6^ cells/well) for 24 h. Then, the medium was removed, and a fresh medium containing different concentrations of compound **19** (1, 2.5, and 5 μM) was added and cultured again for 48 h. Gemcitabine (GEM) was used as a positive control. After culture, the cells were collected and washed twice with cold PBS; binding buffer solution was added to the collected cell precipitate to make the cell concentration reach 1 × 10^6^/mL. Then, 100 μL of the cell suspension was reabsorbed into the new centrifuge tube, and 5 μL Annexin V-FITC and 5 μL PI were added. After incubation at room temperature for 15 min (in a dark place), 400 μL binding buffer was added to each well. Fluorescence of cells was immediately detected with a flow cytometer and used for quantitative analysis.

### Western Blotting

HepG2 cells were seeded in 6-well plates (1 × 10^6^ cells/well) for 24 h. Then, the medium was removed, and a fresh medium containing different concentrations of compound **19** (1, 2.5, and 5 μM) was cultured for another 48 h. The medium was removed and washed with cold PBS; cells were collected and lysed with RIPA buffer, left to stand in an ice bath for 30 min, and centrifuged at 4°C for 20 min at the highest speed. The protein concentration was detected by using the BCA protein assay kit. Proteins were separated by SDS-PAGE gels and transferred to the PVDF membrane. The membrane was blocked with 5% non-fat milk for 2 h. Then, specific primary antibodies were used to bind to the corresponding proteins and left for incubation at 4°C overnight, after washing thrice with TBST (5 min), followed by incubation with a second antibody at room temperature for 50 min. The protein bands were detected using the ECL detection kit, and the gray intensities of the bands were measured by ImageJ software.

### Statistical Analysis

All data are presented as the mean ± SD of at least three independent experiments. The means were compared by one-way ANOVA, followed by Dunnett’s test by GraphPad Prism 8.0 software. When the *p*-value was less than 0.05, the difference between groups was considered statistically significant.

## Results and Discussion

### Structure Elucidation of New Compounds

Compound **1** was afforded as an amorphous white powder. The molecular formula of compound **1** was determined to be C_21_H_34_O_6_ based on its HRESIMS data (*m/z* 427.2318 [M + HCOO]^-^, calcd for C_22_H_35_O_8_ 427.2326), indicating the existence of five degrees of unsaturation. The IR spectrum showed characteristic absorptions attributable to a cyclic olefinic bond (1,638 cm^−1^) and a hydroxyl (3,449 cm^−1^) bond. The ^1^H NMR spectrum (Table 1) of compound **1** revealed the presence of an olefinic proton [*δ*_H_ 5.02 (1H, m)] and three methyl groups [*δ*_H_ 0.80 (3H, s), 0.90 (3H, s), and 1.02 (3H, s)]. The ^13^C NMR and DEPT spectra (Table 2) showed 21 carbon signals due to three methyls, six methylenes, nine methines, and three quaternary carbons. Comprehensive interpretation of the 1D and 2D NMR spectral data allowed full assignment of the ^1^H and ^13^C NMR signals of compound **1** (Table 1 and Table 2). The aforementioned data indicated the presence of a *β*-D-glucopyranosyl moiety (Xiao et al., 2011) [*δ*_C_ 104.8, 78.2, 77.9, 75.2, 71.7, and 62.8]. Acid hydrolysis of compound **1** with 1 mol/L HCl afforded compound **1′** and *β*-D-glucose that was identified by direct comparison with an authentic sample.

**FIGURE 1:**
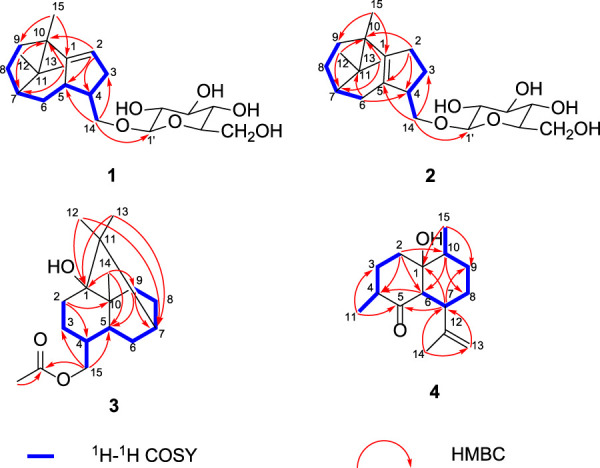
Key ^1^H-^1^H and HMBC correlations.

Analysis of ^1^H–^1^H COSY in combination with HSQC showed correlations from H-2 to H-9, as shown by bold lines in Figure 1. In the HMBC spectrum, as shown in Figure 1, the correlations were observed from H-15 to C-1/C-9, H-2 to C-10/C-5/C-4, H-12 to C-10/C-7, H-13 to C-10/C-7, and H-14 to C-5/C-3/C-1′. Comprehensive interpretation of 1D and 2D NMR spectra of compound **1** revealed the presence of a patchoulene-type sesquiterpene unit in **1**, which was similar to *δ*-patchoulene (Faraldos et al., 2010). However, its carbon spectrum NMR data have not been reported in the literature. The observed key HMBC correlations between H-14 and C-1′, as well as between H-1′ and C-14, indicated that the two units were linked *via* the C-14–O–C-1′ bond to form a patchoulene-type sesquiterpenoid glycoside. Thus, the planar structure of compound **1** was established (Figure 1).

**FIGURE 2:**
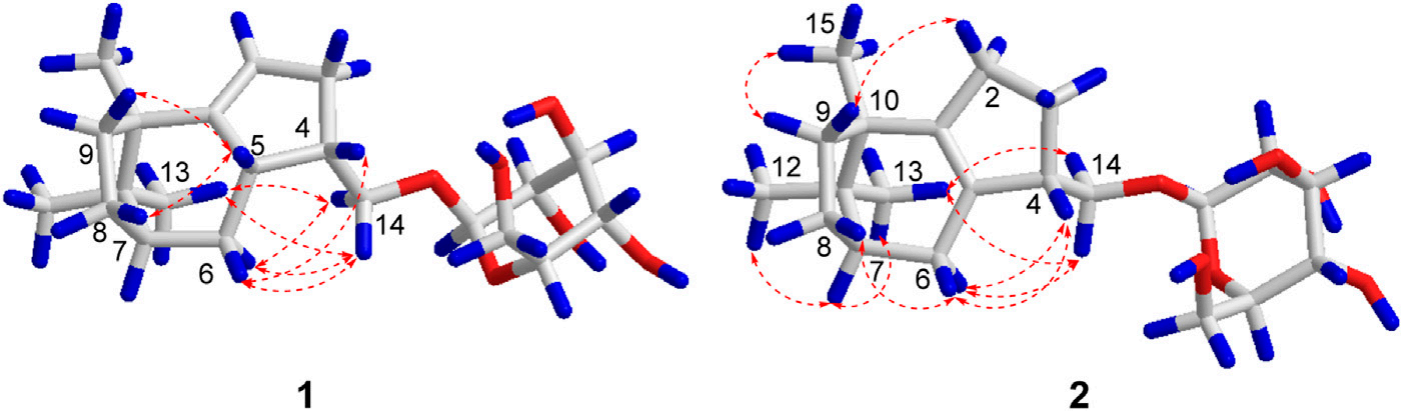
Key NOESY correlations of compounds **1** and **2**.

The relative configuration of compound **1** was established by the NOESY interactions (Figure 2). The correlations between H-14a and H-6a/H-6b and between H-14a/H-14b and H-13 indicated the *β*-orientation of H-4, H-5, H-7, and CH_3_-15. Thus, the relative configuration of the sesquiterpenoid unit in compound **1** was determined.

**FIGURE 3:**
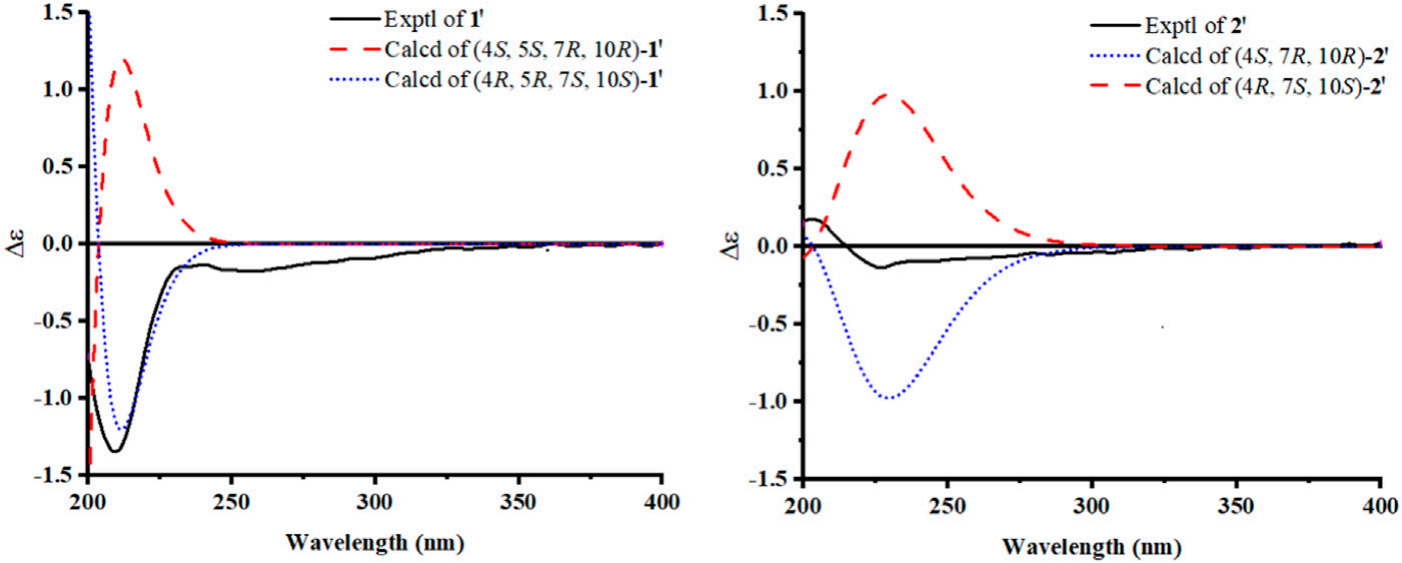
Experimental and calculated CD spectra for compounds **1′** and **2′**.

Finally, according to the quantum chemical electronic circular dichroism (ECD) calculation, as shown in Figure 3, the absolute configuration of compound **1** was deduced to be 4*R*, 5*R*, 7*S*, and 10*S* by a comparison of the experimental ECD spectrum of compound **1′** with the calculated one. Therefore, compound **1** was determined to be (4*R*, 5*R*, 7*S*, 10*S*)-14-hydroxypatchoulene-14-*O*-*β*-D-glucopyranoside, and named Pogopatchoulene A.

Compound **2** was afforded as an amorphous white powder. The molecular formula of compound **2** was deduced to be C_21_H_34_O_6_ by its HRESIMS [M + HCOO]^-^ ion at *m/z* 427.2320 (calcd for C_22_H_35_O_8_ 427.2326), corresponding to five degrees of unsaturation. The UV and IR spectra of compound **2** displayed similar signals to those of **1**. The ^13^C NMR spectrum revealed the presence of 21 carbon signals including three methyls, seven methylenes, seven methines, and four quaternary carbons. The 1D NMR signals of compound **2** closely resembled those of **1**, indicating that they possessed a similar structure except for the presence of a tetrasubstituted double-bond group in compound **2** instead of a trisubstituted double-bond group in compound **1**. A comprehensive comparison of their 2D NMR data revealed that the tetrasubstituted double-bond group in compound **2** was in positions 1 and 5 instead of positions 1 and 2 in compound **1**. Furthermore, compound **2** was hydrolyzed with 1 mol/L HCl to obtain compound **2′** and *β*-D-glucose. Hence, the planar structure of compound **2** was established.

The relative configuration of compound **2** could be suggested by the interpretation of the NOESY data, as shown in Figure 2; the cross peaks between H-14a/H-14b and H-13 and between H-14a and H-6a indicated that H-4, H-7, and CH_3_-15 were in *β*-orientation. Finally, the absolute configuration of compound **2** was determined to be 4*S*, 7*,* and 10*R*, according to the quantum chemical ECD calculation presented in Figure 3. In conclusion, compound **2** was determined as (4*S*, 7*R*, 10*R*)-14-hydroxypatchoulene-14-*O*-*β*-D-glucopyranoside, and named Pogopatchoulene B.

**FIGURE 4:**
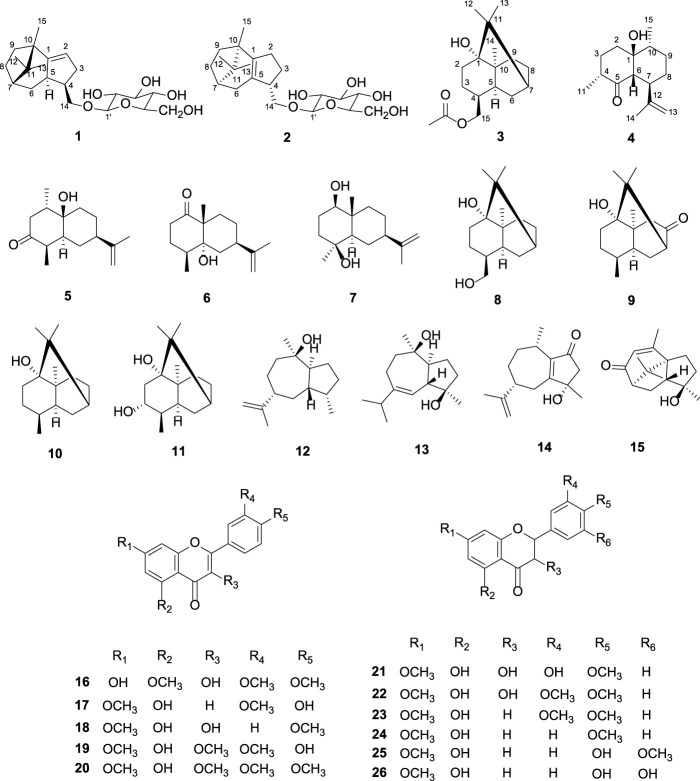
Chemical structures of compounds **1**-**26**.

Compound **3** was shown to have the molecular formula C_17_H_28_O_3_ by its HRESIMS data (*m/z* 281.2109 [M + H]^+^, calcd for C_17_H_29_O_3_: 281.2111). The UV spectrum showed maximal absorption at 286 nm. The IR spectra revealed the characteristic absorptions for hydroxyl (3,449 cm^−1^) and carbonyl (1,685 cm^−1^) bonds. The ^1^H NMR spectrum displayed the signals for four methyl protons [*δ*_H_ 0.84 (3H, s, H-14), 1.07 (6H, overlapped, H-13 and H-12), and 2.04 (3H, s, H-17)]. The ^13^C NMR and DEPT spectra displayed 17 carbon signals including four methyls, six methylenes, three methines, and four quaternary carbons. Comparison of the NMR data of compound **3** with the known compound patchoulan-1, 15-diol **(8)** suggested that their NMR signals were similar except for extra acetyl [*δ*_H_ 2.04 (3H, s), *δ*_C_ 21.1, 171.4] in compound **3**. With the aid of ^1^H-^1^H COSY, HSQC, and HMBC experiments, all the ^1^H and ^13^C NMR signals were assigned, as shown in Table 1 and the relative configuration is shown in Figure 4, which was in accordance with a reaction product named patchouli alcohol acetate (Niwa et al., 1987). However, no relevant nuclear magnetic data on patchouli alcohol acetate had been reported in the literature. Therefore, we reported its carbon spectrum for the first time.

The molecular formula of compound **4** was established as C_15_H_24_O_2_ by a quasi-molecular ion peak at *m/z* 237.1849 [M + H]^+^ (calcd for C_15_H_25_O_2_: 237.1849) in its HRESIMS. IR spectra showed the characteristic absorptions for hydroxyl (3,474 cm^−1^), carbonyl (1707 cm^−1^), and olefinic bonds (1,644 cm^−1^). The ^1^H NMR spectrum displayed two olefinic protons [*δ*_H_ 4.65 (1H, m) and 4.50 (1H, s)] and three methyl groups [*δ*_H_ 1.10 (3H, d), 1.20 (3H, d), and 1.80 (3H, s)]. We compared the NMR data on compound **4** with those of the synthetic compound (+)-[2*R*-(2*α*, 4a*β*, 5*α*, 8*β*, 8a*β*)]-octahydro-4a-hydroxy-2, 5-dimethyl-8-(1-methylethenyl)-1(2H)-naphthalenone (Gijsen et al., 1994), which were exactly the same. Hence, compound **4** was determined.

In a summary, two new patchoulene sesquiterpenoid glycosides (**1–2**), a natural patchoulane-type sesquiterpenoid (**3**) and a natural cadinene-type sesquiterpenoid (**4**) were isolated and determined from *P. cablin*. Other known compounds, as rel-(1*S*, 4*R*, 5*R*, 7*R*, 10*R*)-10-desmethyl-10-hydroxy-1-methyl-3-oxo-11-eudesmene (**5**) (Chavez et al., 1995), corymbolone (**6**) (Garbarino et al., 1985), 1*β*, 4*β*-dihydroxyeudesman-11-ene (**7**) (Li et al., 2005), patchoulan-1, 15-diol (**8**) (Ding et al., 2011), 2, 3, 4, 4*α*, 5, 6, 8, 8*α*-octayhydro-1-hydroxy-4, 8*α*, 9, 9-tetramethyl-1, 6-methanonaphthalen-7(1H)-one (**9**) (Barton et al., 1987), patchouli alcohol (**10**) (Barton et al., 1987), 3*R*-3-hydroxypatchoulol (**11**) (Aleu et al., 2001), pogostol (**12**) (Stierle et al., 2003), 1*S*, 4*R*, 5*S*, 6*R*, 7*S*, 10*S*-1 (5), 6 (7)-diepoxy-4-guaiol (**13**) (Chen et al., 2020), (5*R*, 8*S*)-3-hydroxy-3, 8-dimethyl-5-(prop-1-en-2-yl)-3, 4, 5, 6, 7, 8-hexahydroazulen-1(2H)-one (**14**) (Huang et al., 2015), 8-keto-9 (10)-*α*-patchoulene-4*α*-ol (**15**) (Li et al., 2013), 3,7-dihydroxy-5, 3′, 4′-trimethoxyflavone (**16**) (Dong et al., 1999), velutin (**17**) (Zahir et al., 1996), 3, 5-dihydroxy-7, 4' -dimethoxy flavone (**18**) (Zhang et al., 2001), 5, 4′-dihydroxy-7-methoxyflavone (**19**) (Zhang et al., 2001), 5-hydroxy-3, 7, 3′, 4′-tetramethoxyflavone (**20**) (Wang et al., 2020), (2*R*, 3*R*)-(+)-4′, 7-di-*O*-methyldihydroquercetin (**21**) (Yoon et al., 2019), 7, 3′, 4′-tri-*O*-methyldhq (**22**) (Kiehlmann and Slade, 2003), 5-hydroxy-7, 3′, 4′-trimethoxyflavanone (**23**) (Zhang et al., 2001), 7, 4′-dimethylapigenin (**24**) (Chen et al., 2017), ereiodictyol-7, 3′-dimethyl ether (5, 4′-dihydroxy-7, 3′-dimethoxyflavanone) (**25**) (Vasconcelos et al., 1998), and (2*S*)-5, 3′, 4′ trihydroxy-7-methoxy-flavanone (**26**) (Vasconcelos et al., 1998) were also obtained and established by comparing with reported spectroscopic data in the literature reports. Compounds **5**–**7**, **9**, **13**, **16**, **21**, **22**, and **26** were isolated from this plant for the first time.

### Anti-Proliferative Effects of Isolates

An ethanol crude extract of *P*. *cablin* displayed *in vitro* anti-tumor potential in our preliminary experiments. Herein, most isolates (**1**–**13** and **15**–**26**) were evaluated for antiproliferative activities in HepG2 cells. Among the tested samples, compounds **17** and **19** displayed anti-proliferative effects against HepG2 cells with IC_50_ values of 25.59 and 2.30 μM, respectively. This observation was in agreement with reported data (Huong et al., 2005), in which the IC_50_ value of compound **19** against HepG2 cells was 1.60 μM. Previous studies have revealed that flavonoids with 2 and 3 double bonds have a higher ability to induce cell apoptosis than flavonols (Wang et al., 1999; Hui et al., 2014; Isoda et al., 2014). In addition, the presence of hydroxyl groups at the 4′ positions of the B ring is important for granulocyte differentiation (Takahashi et al., 1998; Isoda et al., 2014). According to our data, flavone compound **19** showed the best antiproliferative effects against HepG2 cells than other flavonoids; it can be concluded that 3-methoxy and 4′- hydroxyl might be important for its activity. The other compounds including sesquiterpenoids and dihydroflavones were inactive, as shown in Table 3. Our data suggest that the anti-tumor effects of *P*. *cablin* might be attributed, at least partially, to its bioactive flavonoids.

**TABLE 3:** Anti-proliferative activity of compounds **1**–**13** and **15–26** against HepG2 cells.

Compound	IC_50_ (μM)	Compound	IC_50_ (μM)
**1**	>50	**15**	>50
**2**	>50	**16**	>50
**3**	>50	**17**	25.59 ± 0.41
**4**	>50	**18**	>50
**5**	>50	**19**	2.30 ± 0.30
**6**	>50	**20**	>50
**7**	>50	**21**	>50
**8**	>50	**22**	>50
**9**	>50	**23**	>50
**10**	>50	**24**	>50
**11**	>50	**25**	>50
**12**	>50	**26**	>50
**13**	>50	—	—

Gemcitabine (GEM) was used as a positive control with an IC_50_ value of 2.32 ± 0.99 μM.

### Compound **19** Induce Apoptosis in HepG2 Cells

**FIGURE 5:**
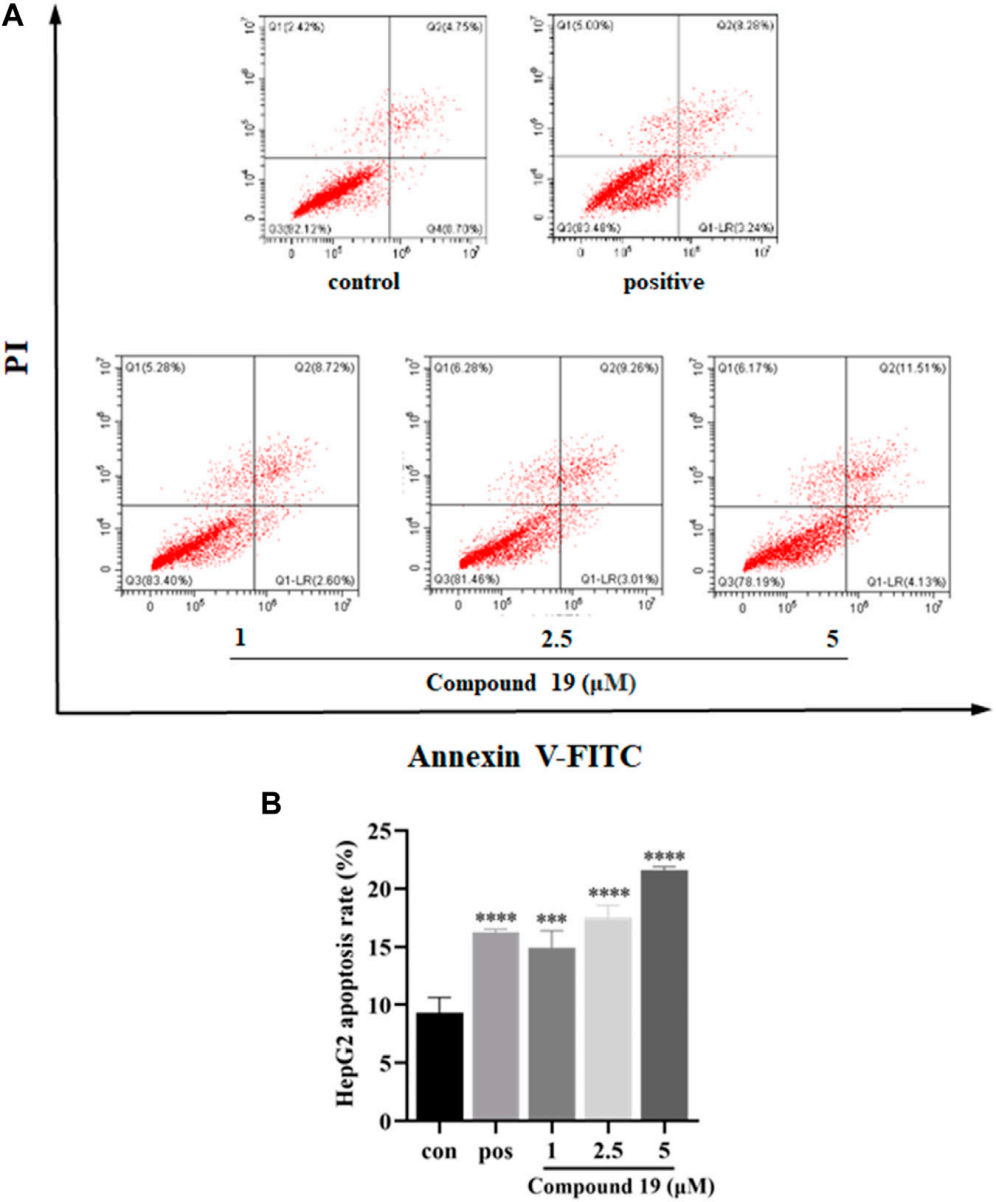
Impact of compound **19** on apoptosis of HepG2 cells. Cells were seeded in 6-well plates (1 × 10^6^ cells/well) and treated with compound **19** (1, 2.5, and 5 μM) for 48 h. Gemcitabine (GEM) was used as a positive control. Annexin V-FITC/PI staining flow cytometry was used to analyze the apoptosis of HepG2 cells after treatment with compound **19**; analysis is shown in **(A)**. The experiments were run in triplicate. Data were presented as mean ± SD. The explicit analysis is shown in **(B)**. ^∗^ *p* < 0.05, ^∗∗^ *p* < 0.01, and ^∗∗∗^ *p* < 0.001 *vs*. the control group.

Apoptosis plays an important role in maintaining tissue homeostasis (Ma, et al., 2018). Induction of apoptosis in tumor cells is an important survival strategy against cancer pathologic progression (Rejhová, et al., 2018), and many natural products have been shown to have anticancer effects through the mitochondrial-mediated apoptosis pathway (Friedman, 2015). The cytotoxic effects of compound **19** on HepG2 have been reported, but the mechanism of action has not been studied. Therefore, the apoptosis-inducing effects of compound **19** in HepG2 cells were also investigated in the current study. As shown in Figure 5, after treatment with compound **19** (1, 2.5, and 5 μM) for 48 h, the flow cytometric apoptosis experiment showed a dose-dependent increase in the percentage of early apoptotic cells when compared with the control group, which showed significant difference (*p* < 0.001).

**FIGURE 6:**
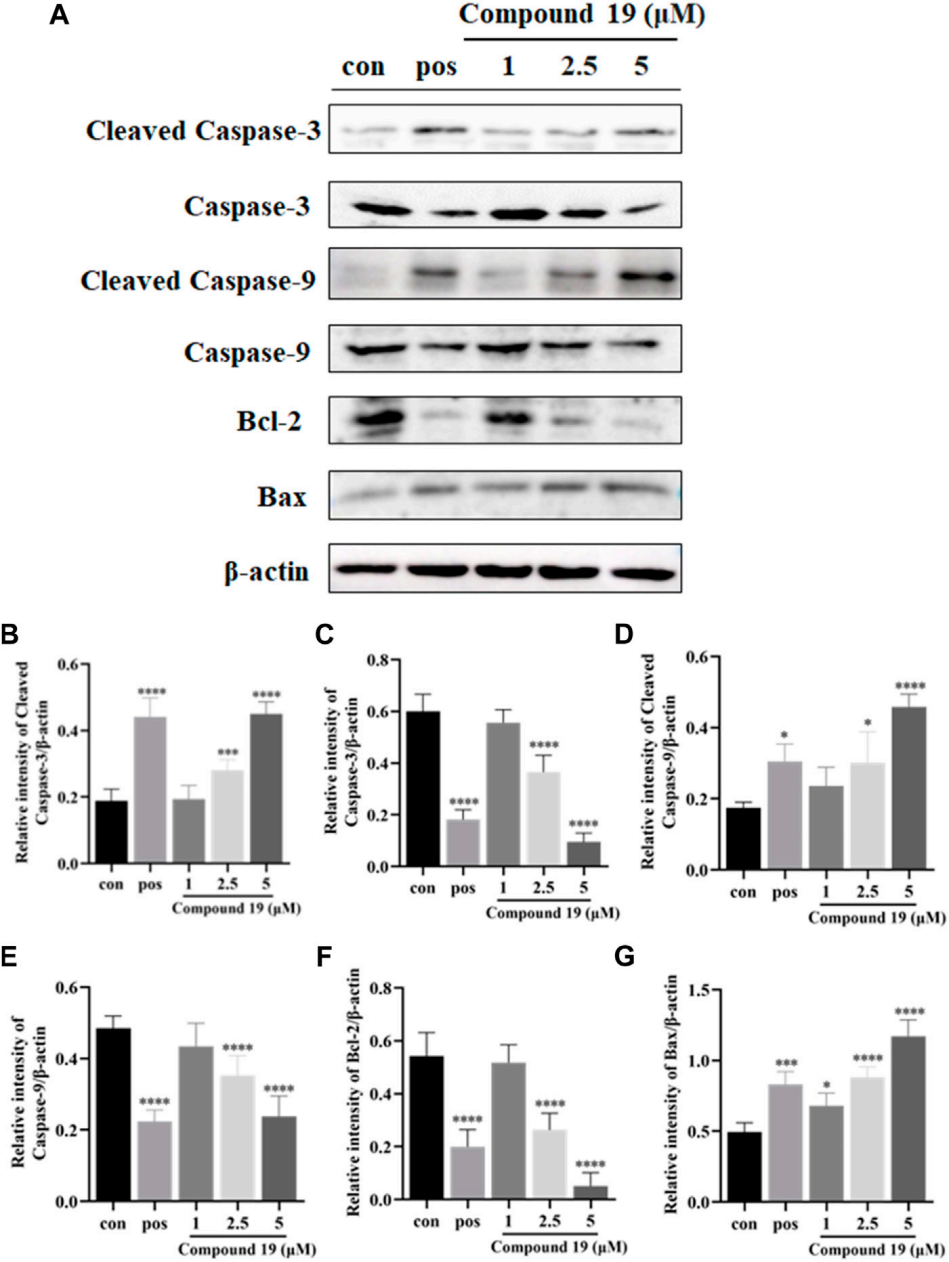
Impact of compound **19** on the protein expressions of cleaved caspase-3, cleaved caspase-9, caspase-3, caspase-9, Bcl-2, and Bax in HepG2 cells. Cells were seeded in 6-well plates (1 × 10^6^ cells/well) and treated with different concentrations of compound **19** (1, 2.5, and 5 μM) for 48 h. Western blotting was used to detect the protein expression levels after compound 19 was applied to HepG2 cells **(A)**. The ratio of the corresponding protein to *β*-actin is calculated in **(B–G)**. The experiments were run in triplicate. Data were presented as mean ± SD. ^∗^ *p* < 0.05, ^∗∗^ *p* < 0.01, and ^∗∗∗^ *p* < 0.001 *vs*. the control group.

Additionally, caspase-9 is an important initiator caspase within the cascade of apoptosis transduction in the mitochondrial-mediated apoptosis pathway. Upon proapoptotic factor release into the cytosol, procaspase-9 is recruited at apoptosome and self-cleaved to caspase-9 and then stimulates downstream executioner caspases, such as caspase-3, which subsequently cause apoptotic cell death (Brentnall et al., 2013; Sadeghi et al., 2019). Our results showed that compared with the control group, compound **19** significantly upregulated the expressions of cleaved caspase-9 and cleaved caspase-3 and significantly downregulated the expressions of caspase-3 and caspase-9. Moreover, both Bcl-2 (an anti-apoptotic protein) and Bax (a proapoptotic protein) belong to the B-cell lymphoma (Bcl-2) family and are critical in the mitochondrial-mediated apoptotic pathway. Western blotting results are shown in Figure 6; compared with the control group, compound **19** significantly upregulated the expressions of Bax and downregulated the expressions of Bcl-2. Thus, compound **19** may induce apoptosis in HepG2 cells and activate the mitochondrial-mediated apoptotic pathway. Our results showed for the first time that compound **19** exerted anti-proliferative effects by inducing apoptotic cell death.

## Conclusion

In conclusion, this study was a phytochemical investigation that explored the chemical profiles and pharmacological properties of active constituents from *P. cablin*. Twenty-six compounds including two new patchoulene sesquiterpenoid glycosides and two natural sesquiterpenoids were isolated from the aerial parts of *P. cablin*, and the absolute configurations of new compounds (**1**–**2**) were established. The antiproliferative activities against the human tumor cell line (HepG2) of the isolated compounds (**1**–**13** and **15**–**26**) were examined by the MTT assay. Compounds **17** and **19** displayed anti-proliferative effects with IC_50_ values of 25.59 and 2.30 μM against HepG2 cells, respectively. In this article, the related mechanism of the anti-proliferative activity of compound **19** has been further studied, and it was found to induce the apoptosis of HepG2 cells by downregulating the ratio of Bcl-2/Bax and upregulating the expressions of cleaved caspase-3 and cleaved caspase-9. Therefore, findings from the current study supported that the chemical constituents from *P. cablin* were multitudinous, which were beneficial for the further fundamental and development research of *P. cablin*.

## Data Availability

The original contributions presented in the study are included in the article/Supplementary Material; further inquiries can be directed to the corresponding authors.

## References

[B1] Aleu J. Hanson J. R. Hernández Galán R. Collado I. G. (2001). Biotransformation of the Fungistatic Sesquiterpenoids Patchoulol, Ginsenol, Cedrol and Globulol by *Botrytis Cinerea* . J. Mol. Catal. B Enzym. 11, 329–334. 10.1016/S1381-1177(00)00014-X

[B2] Barton D. H. R. Bel?il J.-C. Billion A. Boivion J. Lallemand J.-Y. Mergui S. (1987). Functionalisation of Saturated Hydrocarbons. Part 9. Oxidation of Patchouli Alcohol by the ‘gif System’: Isolation and Organoleptic Properties of Three New Ketonic Derivatives. Helv. Chim. Acta 70, 273–280. 10.1002/hlca.19870700202

[B3] Brentnall M. Rodriguez-Menocal L. De Guevara R. L. Cepero E. Boise L. H. (2013). Caspase-9, Caspase-3 and Caspase-7 Have Distinct Roles during Intrinsic Apoptosis. BMC Cell. Biol. 14 (1), 1–9. 10.1186/1471-2121-14-32 23834359 PMC3710246

[B4] Büchi G. Goldman I. M. Mayo D. W. (1966). The Structures of Two Alkaloids from Patchouli Oil. J. Am. Chem. Soc. 88, 3109–3113. 10.1021/ja00965a040

[B5] Chavez J. P. Gottlieb O. R. Yoshida M. (1995). 10-Desmethyl-1-methyl-eudesmanes from *Ocotea Corymbosa* . Phytochemistry 39, 849–852. 10.1016/0031-9422(94)00977-2

[B6] Chen Y. M. Cao N. K. Tu P. F. Jiang Y. (2017). Chemical Constituents from Murraya Euchrestifolia. Zhongguo Zhong Yao Za Zhi 42, 1916–1921. 10.19540/j.cnki.cjcmm.20170228.002j 29090551

[B7] Chen Y. W. Dong F. W. Qin T. L. Zhang F. Wu S. L. Yang M. Y. (2020). Terpenoids from Stems and Leaves of *Aphanamixis Grandifolia* . Chin. J. Exp. Traditional Med. Formulae 26, 168–173. 10.13422/j.cnki.syfjx.20201311

[B8] Dahai W. Yin Z. Zhang Q. Ye W. Zhang X. Zhang J. (2010). Nonvolatile Chemical Constituents from *Pogostemon Cablin* . Cjcmm 35, 2704–2707. 10.4268/cjcmm20102014 21246823

[B9] Ding W.-B. Lin L.-D. Liu M.-F. Wei X.-Y. (2011). Two New Sesquiterpene Glycosides fromPogostemon Cablin. J. Asian Nat. Prod. Res. 13, 599–603. 10.1080/10286020.2011.577424

[B10] Ding W. Liu M. Wei X. Lin L. (2009). Strong Polarity Components of *Pogostemon Cablin* (Blance) Benth. J. Trop. Subtropical Bot. 17, 610–616. 10.3969/j.issn.1005-3395.2009.06.017

[B11] Dong H. Gou Y.-L. Cao S.-G. Chen S.-X. Sim K.-Y. Goh S.-H. (1999). Eicosenones and Methylated Flavonols from Amomumkoenigii. Phytochemistry 50, 899–902. 10.1016/S0031-9422(98)00622-0

[B12] Faraldos J. A. Wu S. Chappell J. Coates R. M. (2010). Doubly Deuterium-Labeled Patchouli Alcohol from Cyclization of Singly Labeled [2-2H1]Farnesyl Diphosphate Catalyzed by Recombinant Patchoulol Synthase. J. Am. Chem. Soc. 132, 2998–3008. 10.1021/ja909251r 20148554

[B13] Feng C. Sui C. Wu H. (1994). Synthesizing Site and Storing Position of Essential Oil in *Pogostemon Cablin* . Chin. Traditional Herb. Drugs 38, 116–119.

[B14] Friedman M. (2015). Chemistry and Anticarcinogenic Mechanisms of Glycoalkaloids Produced by Eggplants, Potatoes, and Tomatoes. J. Agric. Food Chem. 63 (13), 3323–3337. 10.1021/acs.jafc.5b00818 25821990

[B15] Garbarino J. A. Gambaro V. Chamy M. C. (1985). The Structure of Corymbolone, an Eudesmane Sesquiterpenoid Keto-Alcohol from *Cyperus Corymbosus* . J. Nat. Prod. 48, 323–325. 10.1021/np50038a023

[B16] Gijsen H. J. M. Wijnberg J. B. P. A. de Groot A. (1994). Thermal Rearrangement of Bicyclogermacrane-1,8-Dione. Synthesis of Humulenedione and (−)-cubenol, Starting from Natural (+)-Aromadendrene-V. Tetrahedron 50, 4745–4754. 10.1002/chin.19943323510.1016/s0040-4020(01)85013-4

[B17] Hikino H. Ito K. Takemoto T. (1968). Structure of Pogostol. Chem. Pharm. Bull. 16, 1608–1610. 10.1248/cpb.16.1608

[B18] Huang A.-C. Sefton M. A. Sumby C. J. Tiekink E. R. T. Taylor D. K. (2015). Mechanistic Studies on the Autoxidation of α-Guaiene: Structural Diversity of the Sesquiterpenoid Downstream Products. J. Nat. Prod. 78, 131–145. 10.1021/np500819f 25581486

[B19] Huang L. Mu S. Zhang J. Deng B. Song Z. Hao X. (2009). Chemical Constituents from Involatile Moiety of Pogostemon Cablin. Zhongguo Zhong Yao Za Zhi 34, 410–413. 10.3321/j.issn:1001-5302.2009.04.010 19459301

[B20] Hui H. Chen Y. Yang H. Zhao K. Wang Q. Zhao L. (2014). Oroxylin A Has Therapeutic Potential in Acute Myelogenous Leukemia by Dual Effects Targeting PPARγ and RXRα. Int. J. Cancer 134 (5), 1195–1206. 10.1002/ijc.28435 23934681

[B21] Huong D. T. Luong D. V. Thao T. T. Sung T. V. (2005). A New Flavone and Cytotoxic Activity of Flavonoid Constituents Isolated from *Miliusa Balansae* (Annonaceae). Pharmazie 60, 627–629. 10.1002/chin.200550192 16124409

[B22] Isoda H. Motojima H. Onaga S. Samet I. Villareal M. O. Han J. (2014). Analysis of the Erythroid Differentiation Effect of Flavonoid Apigenin on K562 Human Chronic Leukemia Cells. Chemico-Biological Interact. 220, 269–277. 10.1016/j.cbi.2014.07.006 25058688

[B23] Kiehlmann E. Slade P. W. (2003). Methylation of Dihydroquercetin Acetates: Synthesis of 5-O-Methyldihydroquercetin. J. Nat. Prod. 66, 1562–1566. 10.1021/np034005w 14695797

[B24] Kongkathip N. Sam-ang P. Kongkathip B. Pankaew Y. Tanasombat M. Udomkusonsri P. (2009). Development of Patchouli Extraction with Quality Control and Isolation of Active Compounds with Antibacterial Activity. Agric. Nat. Resour. 43, 519–525.

[B25] Li F. Li C.-J. Ma J. Yang J.-Z. Chen H. Liu X.-M. (2013). Four New Sesquiterpenes from the Stems of *Pogostemon Cablin* . Fitoterapia 86, 183–187. 10.1016/j.fitote.2013.03.010 23518259

[B26] Li X. Yang M. Han Y.-F. Gao K. (2005). New Sesquiterpenes fromErigeron Annus. Planta Med. 71, 268–272. 10.1055/s-2005-837827 15770549

[B27] Ma Z.-J. Lu L. Yang J.-J. Wang X.-X. Su G. Wang Z.-l. (2018). Lariciresinol Induces Apoptosis in HepG2 Cells via Mitochondrial-Mediated Apoptosis Pathway. Eur. J. Pharmacol. 821, 1–10. 10.1016/j.ejphar.2017.12.027 29247613

[B28] Niwa H. Hasegawa T. Ban N. Yamada K. (1987). Stereocontrolled Total Synthesis of (±)-norpatchoulenol and Two Metabolites of Patchouli Alcohol, (±)-hydroxy Patchouli Alcohol and the Corresponding (±)-carboxylic Acid. Tetrahedron 43, 825–834. 10.1016/S0040-4020(01)90019-5

[B29] Rejhová A. Opattová A. Čumová A. Slíva D. Vodička P. (2018). Natural Compounds and Combination Therapy in Colorectal Cancer Treatment. Eur. J. Med. Chem. 144, 582–594. 10.1016/j.ejmech.2017.12.039 29289883

[B30] Sadeghi S. Davoodvandi A. Pourhanifeh M. H. Sharifi N. ArefNezhad R. Sahebnasagh R. (2019). Anti-cancer Effects of Cinnamon: Insights into its Apoptosis Effects. Eur. J. Med. Chem. 178, 131–140. 10.1016/j.ejmech.2019.05.067 31195168

[B31] Stephens P. J. Harada N. (2009). ECD Cotton Effect Approximated by the Gaussian Curve and Other Methods. Chirality 22, 229–233. 10.1002/chir.20733 19408332

[B32] Stierle A. A. Stierle D. B. Goldstein E. Parker K. Bugni T. Baarson C. (2003). A Novel 5-HT Receptor Ligand and Related Cytotoxic Compounds from an Acid Mine Waste Extremophile. J. Nat. Prod. 66, 1097–1100. 10.1021/np030044w 12932132

[B33] Takahashi T. Kobori M. Shinmoto H. Tsushida T. (1998). Structure-activity Relationships of Flavonoids and the Induction of Granulocytic- or Monocytic-Differentiation in HL60 Human Myeloid Leukemia Cells. Biosci. Biotechnol. Biochem. 62 (11), 2199–2204. 10.1271/bbb.62.2199 9972240

[B34] Tang S. Zhang X.-T. Ma Y.-B. Huang X.-Y. Geng C.-A. Li T.-Z. (2020). Artemyrianolides A-S, Cytotoxic Sesquiterpenoids from Artemisia Myriantha. J. Nat. Prod. 83, 2618–2630. 10.1021/acs.jnatprod.0c00396 32842729

[B35] Terhune S. J. Hogg J. W. Lawrence B. M. (1973). Cycloseychellene, a New Tetracyclic Sesquiterpene from. Tetrahedron Lett. 14, 4705–4706. 10.1016/S0040-4039(01)87315-9

[B36] Vasconcelos J. M. J. Silva A. M. S. Cavaleiro J. A. S. (1998). Chromones and Flavanones from *Artemisia Campestris* Subsp. *Maritima* . Phytochemistry 49, 1421–1424. 10.1002/chin.19991127010.1016/s0031-9422(98)00180-0

[B37] Wang I.-K. Lin-Shiau S.-Y. Lin J.-K. (1999). Induction of Apoptosis by Apigenin and Related Flavonoids through Cytochrome C Release and Activation of Caspase-9 and Caspase-3 in Leukaemia HL-60 Cells. Eur. J. Cancer 35 (10), 1517–1525. 10.1016/S0959-8049(99)00168-9 10673981

[B38] Wang Z.-H. Li Q. Huang M. Xu P.-F. Yang L.-P. Zhai Y.-Y. (2020). Chemical Constituents of *Callicarpa Macrophylla* . Chem. Nat. Compd. 56, 1125–1127. 10.1007/s10600-020-03243-4

[B39] Xiao H. M. Zu L. B. Li S. P. Wang K. J. Li N. (2011). Chemical Constituents from Dried Fruits of *Rubus Chingii* . Chin. J. Med. Chem. 21, 220–226. 10.1007/s10570-010-9464-0

[B40] Yoon K. D. Lee J.-Y. Kim T. Y. Kang H. Ha K.-S. Ham T.-H. (2019). *In Vitro* and *In Vivo* Anti-hyperglycemic Activities of Taxifolin and its Derivatives Isolated from Pigmented Rice (*Oryzae Sativa* L. Cv. Superhongmi). J. Agric. Food Chem. 68, 742–750. 10.1021/np034005w10.1021/acs.jafc.9b04962 31880937

[B41] Zahir A. Jossang A. Bodo B. Provost J. Cosson J.-P. Sévenet T. (1996). DNA Topoisomerase I Inhibitors: Cytotoxic Flavones from *Lethedon Tannaensis* . J. Nat. Prod. 59 (7), 701–703. 10.1021/np960336f 8759170

[B42] Zhang G. W. Ma X. Q. Su J. Y. (2001). Flavonoids Isolated from *Pogostemon Cablin* (In English). Chin. Traditional Herb. Drugs 32, 870–873. 10.7501/j.issn.0253-2670.2001.10.2001010478

[B43] Zhang Y. Zhou W.-Y. Song X.-Y. Yao G.-D. Song S.-J. (2020). Neuroprotective Terpenoids from the Leaves of *Viburnum Odoratissimum* . Nat. Prod. Res. 34, 1352–1359. 10.1080/14786419.2018.1514400 30417665

