# Supplementary material for: Chemical Constituents With Antiproliferative Activity From Pogostemon cablin (Blanco) Benth
Source: Front Chem. 2022 Jul 15;10:938851. doi: 10.3389/fchem.2022.938851 (PMC9334562; doi:10.3389/fchem.2022.938851)
Supplement: Supplementary file 1 [file DataSheet1.PDF]

Contents

Fig. S1 Two candidate absolute configurations of **1'**......3

Fig. S2 Experimental ECD curves of **1'** (solid black line), and M062X/TZVP//B3LYP/6-31G(d) calculated ECD spectra of (4*S*, 5*S*, 7*R*, 10*R*)-**1'** (dash red line) and (4*R*, 5*R*, 7*S*, 10*S*)-**1'** (dash blue line)......3

Fig. S3 B3LYP/6-31G(d) optimized lowest energy conformers for (4*S*,5*S*,7*R*,10*R*)-**1'**......4

Table S1 Energy (298.15 K) analysis for (4*S*,5*S*,7*R*,10*R*)-**1'**......4

Table S2 Calculated ECD Data for (4*S*,5*S*,7*R*,10*R*)-**1'** in gas phase......5

Fig. S4 Two candidate absolute configurations of **2'**......9

Fig. S5 Experimental ECD curves of **2'** (solid black line), and M062X/TZVP//B3LYP/6-31G(d) calculated ECD spectra of (4*R*, 7*R*, 10*S*)-**2'** (dash red line) and (4*S*, 7*R*, 10*R*)-**2'** (dash blue line)......9

Fig. S6 B3LYP/6-31G(d) optimized lowest energy conformers for (4*S*, 7*R*,10*R*)-**2'**. 10

Table S3 Energy (298.15 K) analysis for (4*S*,7*R*,10*R*)-**2'**......10

Table S4 Calculated ECD Data for (4*S*, 7*R*,10*R*)-**2'** in gas phase......11

Fig. S7 ^{1}H NMR (500 MHz) spectrum of compound **1** in CD_3OD......18

Fig. S8 ^{13}C NMR (125 MHz) spectrum of compound **1** in CD_3OD......18

Fig. S9 HSQC spectrum of compound **1** in CD_3OD......19

Fig. S10 ^{1}H-^{1}H COSYspectrum of compound **1** in CD_3OD......19

Fig. S11 HMBC spectrum of compound **1** in CD_3OD......20

Fig. S12 NOESY spectrum of compound **1** in CD_3OD......20

Fig. S13 HRESIMS of compound **1**......21

Fig. S14 ^{1}H NMR (500 MHz) spectrum of compound **2** in CD_3OD......21

Fig. S15 ^{13}C NMR (125 MHz) spectrum of compound **2** in CD_3OD......22

Fig. S16 HSQC spectrum of compound **2** in CD_3OD......22

Fig. S17 ^{1}H-^{1}H COSYspectrum of compound **2** in CD_3OD......23

Fig. S18 HMBC spectrum of compound **2** in CD_3OD......23

Fig. S19 NOESY spectrum of compound **2** in CD_3OD......24

Fig. S20 HRESIMS of compound **2**......24

Fig. S21 ^{1}H NMR (500 MHz) spectrum of compound **3** in $CDCl_3$......25

Fig. S22 ^{13}C NMR (125 MHz) spectrum of compound **3** in $CDCl_3$......25

Fig. S23 HSQC spe ctrum of compound **3** in $CDCl_3$. 26
Fig. S24 ^1H-^1H COSYspectrum of compound **3** in $CDCl_3$. 26
Fig. S25 HMBC spectrum of compound **3** in $CDCl_3$. 27
Fig. S26 HRESIMS of compound **3**. 27
Fig. S27 ^1H NMR (500 MHz) spectrum of compound **4** in $CDCl_3$. 28
Fig. S28 ^{13}C NMR (125 MHz) spectrum of compound **4** in $CDCl_3$. 28
Fig. S29 HSQC spectrum of compound **4** in $CDCl_3$. 29
Fig. S30 ^1H-^1H COSYspectrum of compound **4** in $CDCl_3$. 29
Fig. S31 HMBC spectrum of compound **4** in $CDCl_3$. 30
Fig. S32 HRESIMS of compound **4**. 30

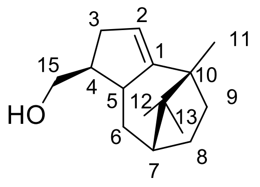

1'

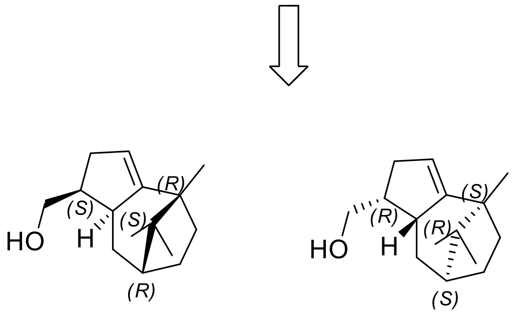

4*S*, 5*S*, 7*R*, 10*R*-**1'** 4*R*, 5*R*, 7*S*, 10*S*-**1'**

Fig. S1 Two candidate absolute configurations of **1'**.

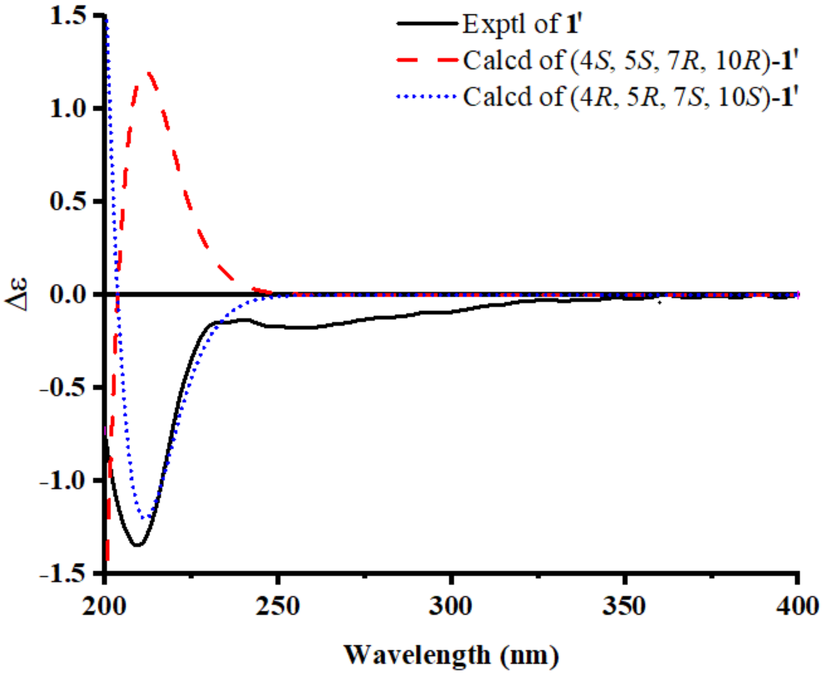

Fig. S2 Experimental ECD curves of **1'** (solid black line), and M062X/TZVP//B3LYP/6-31G(d) calculated ECD spectra of **(4*S*, 5*S*, 7*R*, 10*R*)-1'** (dash red line) and **(4*R*, 5*R*, 7*S*, 10*S*)-1'** (dash blue line).

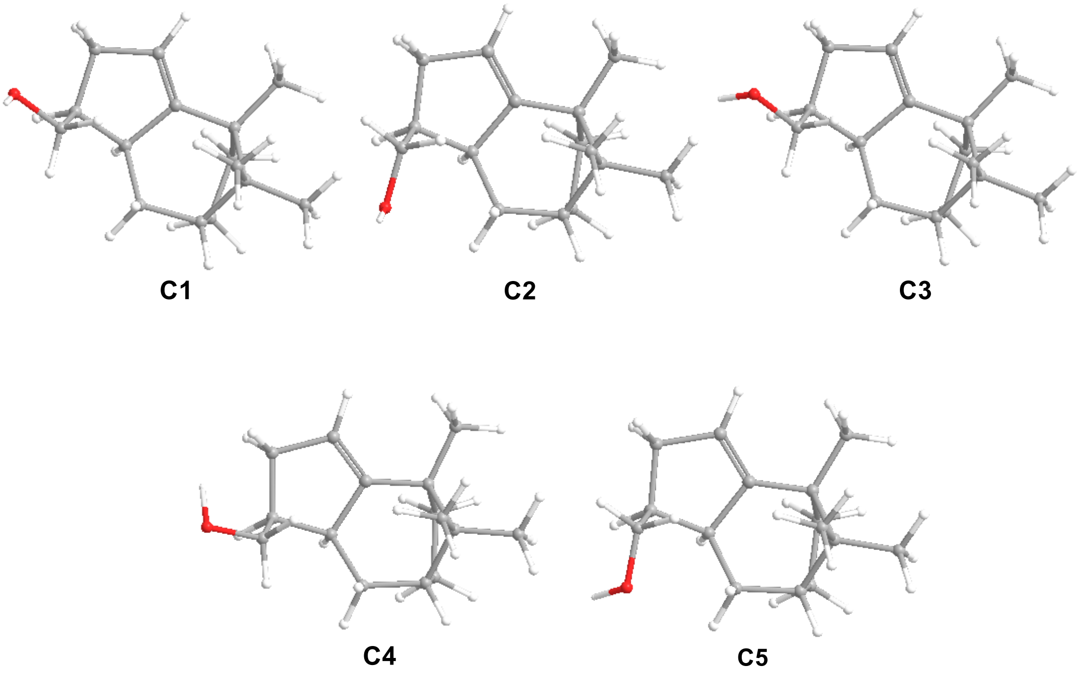

Fig. S3 B3LYP/6-31G(d) optimized lowest energy conformers for **(4*S*,5*S*,7*R*,10*R*)-1'**.

Table S1 Energy (298.15 K) analysis for **(4*S*,5*S*,7*R*,10*R*)-1'**.

	Conf.	G (Hartree)	ΔG (Kcal/mol)	Boltzmann Distribution
	**C1**	-660.921676	0.40725399	0.13792061
	**C2**	-660.921747	0.36270078	0.148697464
**(4*S*,5*S*,7*R*,10*R*)-1'**	**C3**	-660.922325	0	0.274347329
	**C4**	-660.921927	0.24974898	0.179945111
	**C5**	-660.922271	0.03388554	0.259089486

Table S2 Calculated ECD Data for **(4*S*,5*S*,7*R*,10*R*)-1'** in gas phase.

State	C1		C2		C3	
	Excitation energies(eV)	Rotatory Strengths*	Excitation energies(eV)	Rotatory Strengths*	Excitation energies(eV)	Rotatory Strengths*
1	6.666	22.7744	6.6974	46.5813	6.6602	25.3746
2	6.9654	-53.9164	6.9524	-47.4799	6.9582	-66.8482
3	7.2244	-18.0612	7.217	-20.3	7.285	8.1754
4	7.4391	-9.3253	7.3931	-38.9525	7.4442	-1.7655
5	7.6038	-4.145	7.5284	10.8883	7.5976	7.1488
6	7.6179	20.4242	7.5998	-7.962	7.62	-16.1169
7	7.6416	-13.3844	7.7303	12.7449	7.6908	-4.5368
8	8.0299	11.9388	7.9864	-4.3846	8.0056	15.2845
9	8.1527	1.4545	8.0524	9.0396	8.1137	10.5351
10	8.29	14.085	8.251	6.4185	8.3227	5.471
11	8.391	61.2609	8.3308	-12.4247	8.3753	28.3496
12	8.4512	12.2052	8.4425	32.1648	8.4109	4.5359
13	8.4857	8.2935	8.4551	8.4738	8.5082	-0.3603
14	8.5306	-13.0181	8.5477	1.111	8.5274	-13.9242
15	8.5632	-13.4297	8.5779	31.2809	8.5905	10.3183
16	8.6016	-15.4242	8.6791	-38.8089	8.6622	-3.002
17	8.658	12.9464	8.712	1.4679	8.6825	-0.8206
18	8.7486	10.8993	8.7763	-71.4645	8.7067	3.8804
19	8.799	-22.3287	8.8009	4.1738	8.8028	-4.0728
20	8.8192	5.1012	8.896	17.486	8.8327	5.3989
21	8.8941	2.5855	8.9394	8.4588	8.852	-32.6084
22	8.926	-22.964	8.9474	-12.4887	8.887	-8.4203
23	8.9853	-1.0744	9.0008	13.993	8.9441	-24.2248
24	9.0229	24.5803	9.0163	-6.2269	8.99	-32.9965
25	9.101	-7.3113	9.0491	-21.5855	9.0365	-11.7101
26	9.1326	-8.456	9.0804	-33.862	9.1044	-18.5773

27	9.1787	-0.4494	9.09	2.0176	9.1393	1.3467
28	9.1893	-1.5634	9.1386	-10.586	9.1695	28.3212
29	9.227	-51.9226	9.1968	6.2335	9.183	4.8286
30	9.2496	6.3816	9.2043	3.7791	9.2036	6.5077
31	9.2734	-19.7373	9.2172	9.7873	9.2444	10.4862
32	9.2966	-58.217	9.2901	-4.723	9.2768	-3.3015
33	9.3421	-7.8654	9.3236	7.9181	9.3227	-49.5815
34	9.3838	-21.3796	9.3382	-16.6623	9.3324	-2.5873
35	9.4045	-0.4624	9.3733	3.7908	9.382	1.7616
36	9.4143	2.7542	9.42	11.0411	9.4244	-12.5084
37	9.4583	16.85	9.4559	-23.4021	9.4545	-15.3564
38	9.4684	-31.1715	9.4778	26.988	9.4753	5.6614
39	9.5184	47.1966	9.4792	19.726	9.5158	49.0615
40	9.5365	20.1528	9.517	-36.7048	9.546	-20.2345
41	9.5579	17.3741	9.5411	11.5791	9.5571	-1.4777
42	9.5738	-10.8131	9.5631	-0.6968	9.5743	13.3703
43	9.5942	-39.1642	9.5836	27.5057	9.6062	4.0572
44	9.6487	-1.8449	9.6155	-28.5826	9.6257	-16.7489
45	9.6826	38.3988	9.6599	-9.648	9.6396	24.7355
46	9.6964	-4.7193	9.6695	51.0349	9.6582	33.8525
47	9.7392	-24.249	9.6929	28.3239	9.7304	-63.8165
48	9.7445	89.1624	9.7023	-1.7544	9.7487	32.6538
49	9.7881	28.2266	9.7433	1.1337	9.7779	12.6117
50	9.8022	35.2047	9.7783	4.571	9.7822	-14.0454
51	9.8079	-27.6273	9.7969	48.4731	9.8031	21.5755
52	9.8208	48.7574	9.8129	2.1759	9.8365	0.2955
53	9.8511	4.5286	9.845	64.7474	9.8619	68.0203
54	9.8638	-9.3727	9.8469	6.5176	9.8697	50.2228
55	9.872	58.9683	9.8655	5.0805	9.8767	23.9938
56	9.8939	59.0803	9.8892	-57.4587	9.9245	-32.998

57	9.9149	-0.3156	9.9262	-13.0787	9.9411	8.6549
58	9.9415	-27.6452	9.9388	-45.0081	9.9775	20.9653
59	9.9458	-21.4406	9.959	-59.5888	9.9781	26.6445
60	9.9753	-12.1669	9.983	-49.1528	9.9913	21.6587

State	**C4**		**C5**	
	Excitation energies(eV)	Rotatory Strengths*	Excitation energies(eV)	Rotatory Strengths*
1	6.6671	18.7324	6.6735	38.9405
2	6.9327	-44.2769	6.945	-56.6886
3	7.2494	-29.8346	7.1976	5.8961
4	7.5432	34.5906	7.4655	-38.9989
5	7.5729	-25.5281	7.5252	17.1208
6	7.6157	0.245	7.6088	-14.4553
7	7.8228	-14.6732	7.782	-10.6595
8	7.9795	12.8779	7.962	7.8015
9	8.1785	-5.4538	8.007	5.1467
10	8.2902	10.654	8.2316	-15.9107
11	8.381	14.7257	8.3056	11.7763
12	8.506	32.9498	8.4324	-12.6616
13	8.5425	9.6918	8.4664	46.3141
14	8.5843	-2.5963	8.5177	-9.7559
15	8.6225	15.3438	8.5881	19.4091
16	8.6726	-17.2855	8.6712	12.8558
17	8.7101	-4.5448	8.6916	19.4817
18	8.7343	-4.0945	8.7553	-106.2195
19	8.8095	-7.4852	8.8122	0.3031
20	8.8516	-29.1395	8.8391	-7.4804
21	8.8934	-7.2438	8.8546	11.181
22	8.9348	2.2278	8.9178	0.0914
23	8.9734	-38.6519	8.9522	-25.947
24	8.989	26.1223	8.9988	-4.5334
25	9.0327	-7.7456	9.0368	-39.4034
26	9.1092	-10.8909	9.0565	13.231
27	9.1323	-11.8327	9.0804	-4.6768
28	9.1549	-13.2591	9.1329	-10.7273
29	9.2037	-3.9154	9.1891	5.9114
30	9.2245	-11.8629	9.2239	-0.5406
31	9.2639	6.5089	9.2404	-4.2378
32	9.3373	20.0868	9.2777	-2.1073
33	9.3453	8.2677	9.3282	-14.2813

34	9.3909	-11.0646	9.3591	-1.0829
35	9.4032	-27.3412	9.4053	28.6456
36	9.4365	65.9056	9.4376	-31.1279
37	9.4732	-39.9691	9.4529	-45.9703
38	9.4842	-21.9917	9.4701	11.4117
39	9.5168	-6.0798	9.4923	8.4337
40	9.5365	-1.7747	9.513	5.184
41	9.543	-7.3135	9.5316	31.1958
42	9.5773	47.3278	9.5624	-19.6366
43	9.6025	-26.9756	9.5829	-0.8884
44	9.639	32.3686	9.6087	42.0102
45	9.6485	-2.8891	9.6257	12.3896
46	9.671	5.9773	9.6372	-6.9106
47	9.7121	-14.7678	9.6691	34.0538
48	9.7319	38.6016	9.6798	0.4153
49	9.776	0.9671	9.7039	66.4757
50	9.8108	23.9994	9.7604	5.9638
51	9.8385	-8.5352	9.7761	-6.5401
52	9.8553	59.9992	9.8108	-47.3658
53	9.8652	-51.0995	9.8192	-23.7142
54	9.8997	15.4094	9.8347	36.3396
55	9.9303	43.7372	9.8663	29.2878
56	9.9456	-65.4569	9.8796	-48.1889
57	9.9541	56.4753	9.9168	-23.7241
58	9.9709	17.4176	9.9226	34.6672
59	9.9889	-16.3657	9.9438	-8.6206
60	10.0237	30.9787	9.9981	-16.8718

* R(velocity)   10**-40 erg-esu-cm

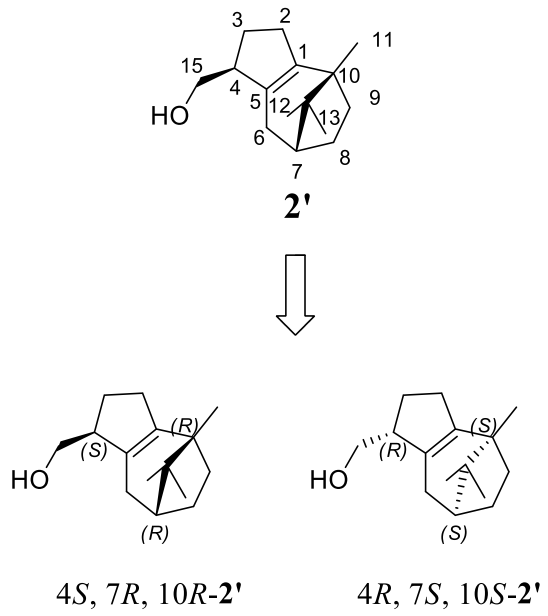

Fig. S4 Two candidate absolute configurations of **2'**.

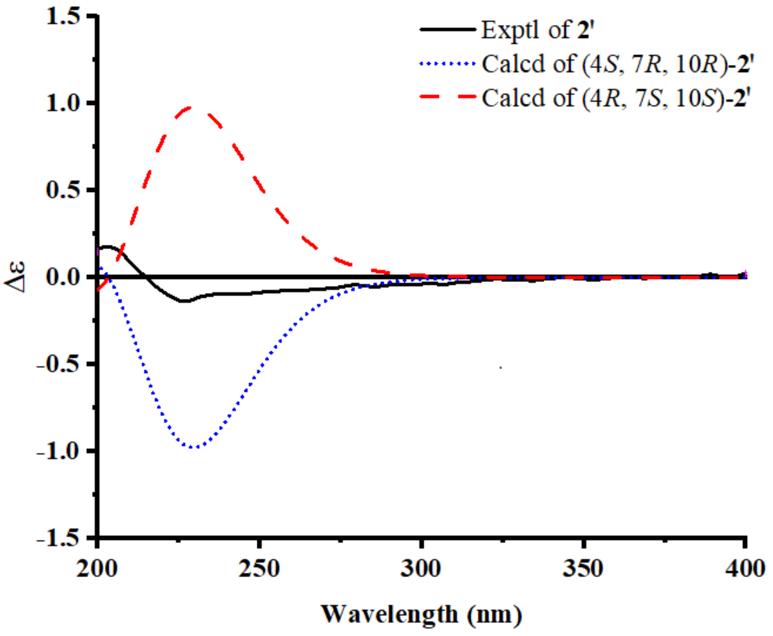

Fig. S5 Experimental ECD curves of **2'** (solid black line), and M062X/TZVP//B3LYP/6-31G(d) calculated ECD spectra of **(4*R*, 7*R*, 10*S*)-2'** (dash red line) and **(4*S*, 7*R*, 10*R*)-2'** (dash blue line).

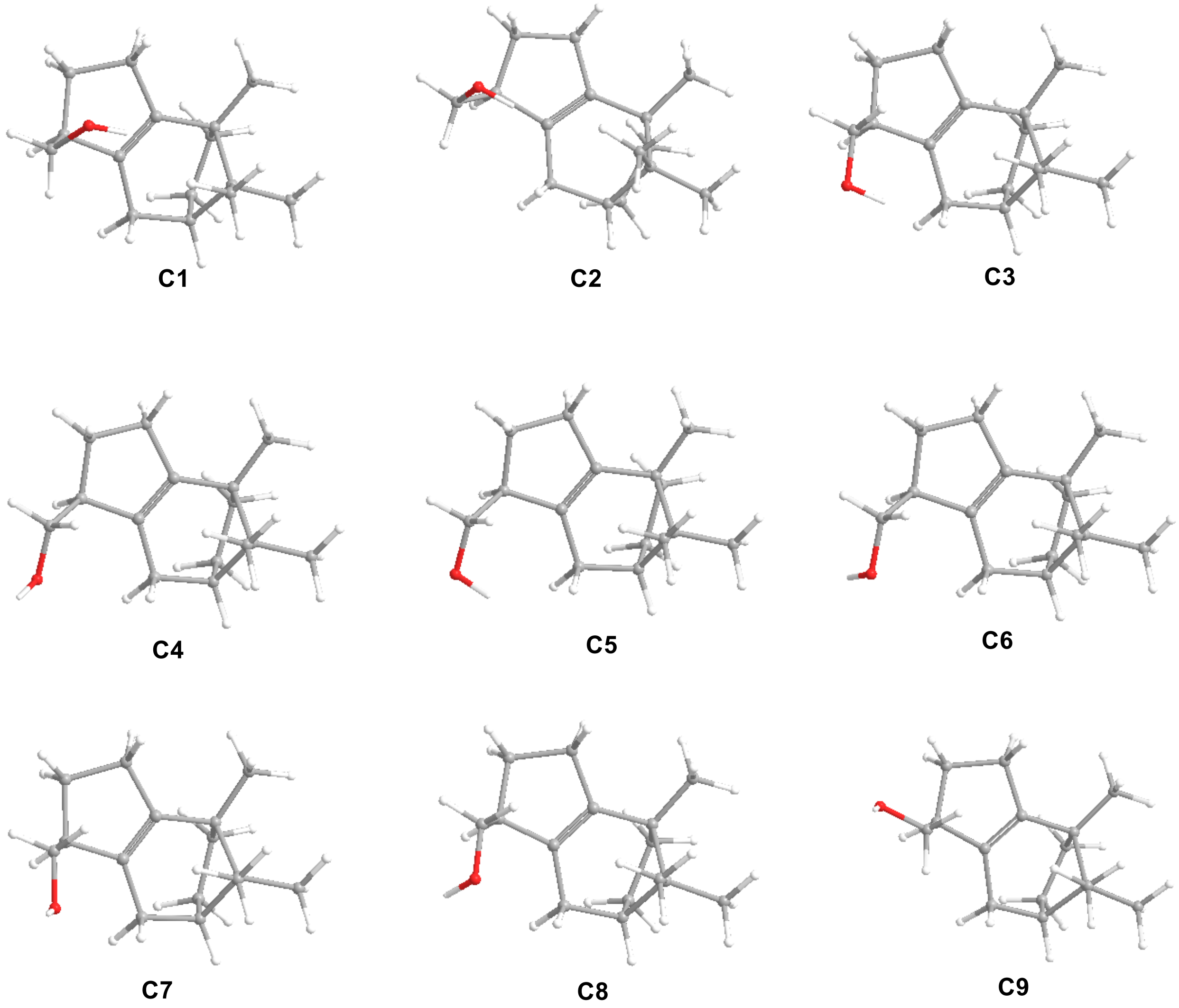

Fig. S6 B3LYP/6-31G(d) optimized lowest energy conformers for **(4*S*, 7*R*,10*R*)-2'**.

Table S3 Energy (298.15 K) analysis for **(4*S*,7*R*,10*R*)-2'**.

	Conf.	G (Hartree)	ΔG (Kcal/mol)	Boltzmann Distribution
	**C1**	-660.932598	0.3514056	0.204846124
**(4*S*,7*R*,10*R*)-2'**	**C2**	-660.933158	0	0.370801323
	**C3**	-660.93125	1.19728908	0.049099207

	**C4**	-660.931341	1.14018567	0.054069572
	**C5**	-660.931287	1.17407121	0.051062489
	**C6**	-660.931965	0.74861943	0.104741654
	**C7**	-660.93109	1.29769068	0.041442128
	**C8**	-660.931752	0.88227906	0.083578822
	**C9**	-660.931065	1.31337843	0.040358682

Table S4 Calculated ECD Data for **(4*S*, 7*R*,10*R*)-2'** in gas phase.

State	**C1**		**C2**		**C3**	
	Excitation energies(eV)	Rotatory Strengths*	Excitation energies(eV)	Rotatory Strengths*	Excitation energies(eV)	Rotatory Strengths*
61	6.3441	-17.0893	6.3791	-44.8204	6.2267	-82.0404
62	6.5037	-2.0689	6.4388	-5.7769	6.3445	29.9508
63	7.289	-3.6803	7.1283	46.1574	6.7965	35.1065
64	7.3617	-12.0934	7.3414	-12.7517	7.2521	-23.2029
65	7.4919	-0.8417	7.3669	-2.1508	7.4021	19.3938
66	7.5708	-20.0914	7.5086	7.1418	7.4985	1.4405
67	7.6582	8.3188	7.6184	-4.2717	7.6148	7.7213
68	7.7741	5.2622	7.6675	-11.2874	7.676	1.0013
69	7.9434	9.499	8.0143	25.3139	7.9293	-2.8915
70	8.045	0.2882	8.044	5.462	7.9827	29.3411
71	8.0935	13.1722	8.1464	-7.0579	8.1636	5.5863
72	8.388	12.1049	8.2414	-2.6427	8.2443	-11.1187
73	8.4074	-1.2455	8.39	-6.3553	8.3285	12.4072
74	8.4241	15.1451	8.4336	20.2286	8.3569	-4.2005
75	8.4424	-5.7457	8.4715	-5.2156	8.3906	-18.3205
76	8.5079	-3.4671	8.4944	8.038	8.4551	-8.457

77	8.5356	5.2574	8.5367	-11.916	8.5324	30.1154
78	8.663	-1.9318	8.6347	2.6423	8.6417	-25.2573
79	8.7163	3.0405	8.7994	17.9057	8.7229	33.4917
80	8.8878	-8.1753	8.8841	54.8076	8.7634	-36.993
81	8.9167	25.8422	8.9183	-20.8154	8.8195	-7.2285
82	8.9493	60.2437	8.9414	2.4548	8.8642	9.9308
83	8.9617	-41.4887	8.9471	21.4942	8.9026	-7.401
84	9.0015	17.1175	8.9715	21.6596	8.9355	-0.1661
85	9.0443	-38.1957	9.0237	-45.5595	8.9944	8.7433
86	9.0828	4.7482	9.0624	-4.6943	9.0193	3.3572
87	9.1102	-0.1912	9.0829	12.5396	9.0295	9.5188
88	9.123	5.9668	9.1297	9.6223	9.0396	26.1755
89	9.1484	2.4058	9.1642	14.4848	9.0795	-2.1688
90	9.2068	63.4421	9.1989	3.9393	9.1169	-13.967
91	9.2421	-10.2756	9.2347	1.19	9.1657	12.8192
92	9.2854	-9.3955	9.2685	-2.5498	9.1802	1.3882
93	9.2978	-9.684	9.2879	31.1449	9.2117	-4.9925
94	9.3177	-7.9088	9.2941	0.4083	9.2584	-19.6803
95	9.335	-4.648	9.323	-23.5786	9.2673	-17.9116
96	9.3411	11.4176	9.3308	-9.222	9.2727	-37.5736
97	9.3732	16.9452	9.3579	-35.5551	9.3156	8.2866
98	9.4159	7.8154	9.3996	-7.3435	9.3409	30.9361
99	9.4461	7.2327	9.4202	-6.1554	9.3696	2.1673
100	9.5021	7.2766	9.4535	-24.7422	9.4174	-1.7758
101	9.508	-4.609	9.4716	-32.8821	9.4635	13.4711
102	9.5478	-70.7856	9.4954	-14.5241	9.5105	-73.3759
103	9.5755	4.4794	9.5153	1.7278	9.5252	0.4333
104	9.6027	9.0168	9.5373	-23.0789	9.5987	26.9721
105	9.6444	-18.6252	9.5694	15.3751	9.6124	2.2392
106	9.6638	4.0738	9.6305	-17.7026	9.6379	-18.7231

107	9.6787	-49.7942	9.6928	-26.7585	9.6437	24.6618
108	9.6949	-14.0203	9.7192	19.2775	9.6948	-32.6977
109	9.7242	-32.3084	9.7416	-11.2394	9.721	10.2166
110	9.7784	18.691	9.7709	-39.4635	9.7271	-56.2709
111	9.8193	-0.3401	9.7897	-8.3736	9.7433	8.5319
112	9.8354	31.5059	9.8	24.3689	9.7887	29.1999
113	9.8551	2.2756	9.826	16.3903	9.8011	-0.8578
114	9.8635	-34.4631	9.8614	4.0817	9.8334	-0.2314
115	9.8707	35.5959	9.8726	17.955	9.8659	-19.3291
116	9.8946	9.9008	9.9051	8.3531	9.8751	23.8134
117	9.9114	-11.9199	9.9197	34.9857	9.8882	32.922
118	9.9138	-1.0052	9.9333	-26.9509	9.8999	-0.9426
119	9.9453	-31.3768	9.9423	5.5481	9.9311	8.8385
120	9.9869	30.7862	9.9606	-16.6285	9.9438	-37.098

State	**C4**		**C5**		**C6**	
	Excitation energies(eV)	Rotatory Strengths*	Excitation energies(eV)	Rotatory Strengths*	Excitation energies(eV)	Rotatory Strengths*
1	6.3531	-34.6915	6.35	-50.5408	6.2951	-49.3693
2	6.4951	-17.8409	6.4906	-12.6275	6.4501	-2.1441
3	7.0151	40.6108	6.9931	50.5393	6.9639	-15.5872
4	7.2152	-8.7519	7.2793	11.2723	7.2338	37.8143
5	7.2963	3.6901	7.4831	14.3721	7.3263	-1.7042
6	7.4954	-6.6645	7.5278	-2.3981	7.4588	-8.1386
7	7.5393	3.9108	7.5925	-8.5534	7.5392	2.9219
8	7.5579	2.4735	7.6711	4.7115	7.6496	2.674
9	7.6767	0.3869	7.8185	-13.2494	7.745	6.6076
10	7.9741	24.5811	8.0183	18.621	7.9594	27.0977
11	8.1509	-2.6309	8.235	-4.1333	8.1124	8.17
12	8.2346	10.9323	8.2954	0.8983	8.2793	-2.3938
13	8.3818	1.3893	8.3261	1.1335	8.3547	-8.1227

14	8.4204	13.2534	8.4145	3.5512	8.4062	10.1937
15	8.4391	-46.217	8.4801	4.1816	8.4315	0.2081
16	8.4674	18.3349	8.4954	-20.2821	8.4869	4.6149
17	8.6634	19.4652	8.5669	6.7743	8.6135	-23.6775
18	8.6825	-24.5726	8.6789	-3.1791	8.6751	-1.9652
19	8.7423	18.6252	8.7302	-34.0991	8.734	-0.5774
20	8.8092	26.9279	8.7483	21.1025	8.7561	33.301
21	8.8781	3.6909	8.881	25.1644	8.8534	32.752
22	8.9349	8.1497	8.9251	-43.2744	8.8711	8.2648
23	8.9672	18.2664	8.9422	3.02	8.9002	14.3709
24	8.9935	-57.4304	8.9689	22.5665	8.9313	26.4491
25	9.0264	0.7169	9.0388	22.5304	9.0137	-67.0449
26	9.0599	-7.9312	9.0805	-30.7251	9.0415	41.4552
27	9.082	3.726	9.088	34.3103	9.056	-10.4248
28	9.1085	34.306	9.1086	-9.2898	9.0793	11.6729
29	9.1162	9.5405	9.1172	5.4161	9.1085	-3.9124
30	9.1898	0.0277	9.1994	-26.1222	9.1406	-12.9243
31	9.1998	28.1344	9.2259	-0.15	9.1579	-9.8782
32	9.2087	-10.5329	9.2489	-6.6786	9.206	-0.8952
33	9.2419	-11.6309	9.2678	-25.0902	9.2404	0.178
34	9.2468	-15.7867	9.2821	23.0007	9.2668	-16.8251
35	9.3013	-8.831	9.2866	-13.2358	9.2783	15.3786
36	9.3072	-5.6303	9.3464	0.3069	9.3305	-21.8334
37	9.3666	-13.8133	9.3791	16.7507	9.3566	-7.4584
38	9.3842	14.6456	9.4066	-10.316	9.3888	-18.6641
39	9.3937	-14.056	9.4368	9.1486	9.4007	2.2335
40	9.4501	-4.9038	9.4504	-24.0379	9.4478	-3.3999
41	9.4809	4.4356	9.4695	25.0424	9.4566	7.6681
42	9.5418	0.6567	9.5188	-0.2896	9.4866	6.7491
43	9.5667	-26.5497	9.5427	-49.3177	9.506	19.2941

44	9.5718	-40.5079	9.5848	14.3616	9.5471	6.1957
45	9.5811	-41.7435	9.5936	-20.2917	9.568	-10.5108
46	9.5972	2.3891	9.6165	13.479	9.5983	5.1406
47	9.6294	10.5554	9.6593	-6.6222	9.6235	-24.7821
48	9.6435	-18.0158	9.6841	16.4886	9.6619	-32.31
49	9.6555	3.7331	9.7236	-59.4043	9.6919	7.4729
50	9.6763	-3.1795	9.731	25.1908	9.7084	22.5405
51	9.7179	-1.3999	9.7404	19.0819	9.7318	7.4673
52	9.7632	7.1506	9.782	-12.7681	9.7502	-1.9788
53	9.7673	-46.9139	9.8041	-21.6845	9.7652	33.7311
54	9.8031	40.4803	9.8291	47.3122	9.7977	-5.3029
55	9.8069	-6.6282	9.8609	14.7152	9.8518	-15.8669
56	9.8327	9.2587	9.8889	-7.674	9.8554	60.7865
57	9.8556	75.5324	9.8958	7.019	9.8585	-55.4269
58	9.8757	2.569	9.9126	20.4981	9.8823	9.5837
59	9.8892	-23.8869	9.9412	2.4781	9.9079	16.2304
60	9.9129	-4.9958	9.9648	53.301	9.9121	-22.6595

State	**C7**		**C8**		**C9**	
	Excitation energies(eV)	Rotatory Strengths*	Excitation energies(eV)	Rotatory Strengths*	Excitation energies(eV)	Rotatory Strengths*
1	6.3318	-40.3352	6.2296	-52.0539	6.399	-6.2435
2	6.417	-0.307	6.4201	-0.3365	6.514	-19.0577
3	6.9617	28.6309	6.8844	8.0655	7.1514	-18.6151
4	7.2185	-6.0293	7.1871	-1.3703	7.2627	2.9679
5	7.2821	-22.2695	7.3621	-10.6025	7.2985	3.9135
6	7.4931	4.8666	7.4909	-2.5301	7.4265	0.2286
7	7.5563	1.6814	7.6432	9.993	7.6075	12.9612
8	7.714	9.1153	7.714	-7.411	7.7593	-22.5063
9	7.7499	-3.9138	7.7724	16.3747	7.7733	10.4377

10	7.9333	26.4185	7.8973	27.2252	8.014	26.999
11	8.111	-12.1858	8.1504	0.4339	8.089	-3.9911
12	8.3008	-2.5906	8.2802	10.1169	8.3147	-2.0304
13	8.3518	17.337	8.3014	-3.8943	8.3448	28.0034
14	8.3861	4.698	8.3618	42.205	8.4004	13.8282
15	8.4148	-26.0275	8.4498	-13.2844	8.4447	-12.4723
16	8.4803	3.1983	8.5045	-12.0985	8.4705	-4.5206
17	8.5348	24.897	8.5823	-51.3856	8.5512	12.3836
18	8.6291	-27.2021	8.6154	6.2659	8.6049	-21.0783
19	8.7142	6.7996	8.7266	10.4691	8.6825	16.5916
20	8.7992	17.1285	8.756	-7.0801	8.758	5.8582
21	8.9001	2.6169	8.8411	-4.8743	8.8073	3.6592
22	8.9257	-20.3772	8.8997	-1.6784	8.9642	-13.9861
23	8.9503	-29.0413	8.9111	35.5246	8.9987	-36.8606
24	8.9931	4.1444	8.9487	7.2065	9.0244	5.9408
25	9.0111	16.4639	8.9997	-41.9398	9.0502	3.7391
26	9.0268	-16.5216	9.0458	5.2327	9.0807	5.9574
27	9.0366	23.1299	9.0663	28.1752	9.0914	-28.2865
28	9.0985	-7.018	9.0884	13.3895	9.1202	4.3089
29	9.1122	5.0681	9.1088	10.8397	9.1656	57.3127
30	9.1294	38.1106	9.1251	32.2791	9.1748	-5.1101
31	9.161	18.3004	9.184	-11.3646	9.2585	-24.6243
32	9.2068	-1.6825	9.2039	-0.4898	9.2727	-7.4441
33	9.2419	-27.1325	9.2232	-1.7888	9.2803	36.6485
34	9.2696	4.4527	9.2438	-5.2397	9.2952	2.4358
35	9.2837	5.2797	9.2706	1.9015	9.3327	22.6257
36	9.2886	-7.4209	9.2786	4.8801	9.3432	29.7641
37	9.2946	2.6128	9.2895	-5.5666	9.4028	5.9756
38	9.3572	-41.4965	9.3426	-13.8358	9.4315	-3.358
39	9.4111	22.7508	9.3959	-6.2597	9.4423	10.1841

40	9.431	6.726	9.4302	7.4561	9.4572	18.3362
41	9.507	0.0814	9.4931	12.4061	9.4742	-18.2689
42	9.5484	-27.6535	9.5176	-26.312	9.4944	-48.8863
43	9.5519	-13.2161	9.5442	-2.4021	9.5139	27.3464
44	9.58	17.8418	9.5618	-30.8198	9.5403	-16.8386
45	9.592	31.3373	9.5999	21.0005	9.5912	-7.0609
46	9.6086	-9.5452	9.6159	-40.8536	9.5976	-21.2153
47	9.6251	-1.7624	9.6249	0.4779	9.6102	0.4731
48	9.6488	-14.2748	9.6385	-0.3395	9.6539	-6.8283
49	9.6635	15.6734	9.6716	-14.482	9.6903	-40.7544
50	9.6898	-26.9791	9.7031	16.7185	9.7414	-77.3396
51	9.7345	-60.6406	9.744	-3.4765	9.7876	33.8598
52	9.7441	-4.4025	9.7544	19.2917	9.8008	-21.1646
53	9.7481	-9.5346	9.7754	23.5945	9.8104	-14.3706
54	9.7959	17.3428	9.8087	21.6509	9.827	-3.532
55	9.8051	9.4007	9.8413	73.1478	9.8342	83.3667
56	9.8518	83.2438	9.8652	-57.3487	9.8739	-11.8612
57	9.8581	-29.3765	9.8725	-0.0735	9.8824	20.3488
58	9.881	3.3156	9.8963	-9.0243	9.8867	28.4165
59	9.8945	14.4544	9.9031	-4.4062	9.93	-40.5186
60	9.9276	-25.3944	9.9278	52.9896	9.9517	-45.1941

* R(velocity) 10**-40 erg-esu-cm

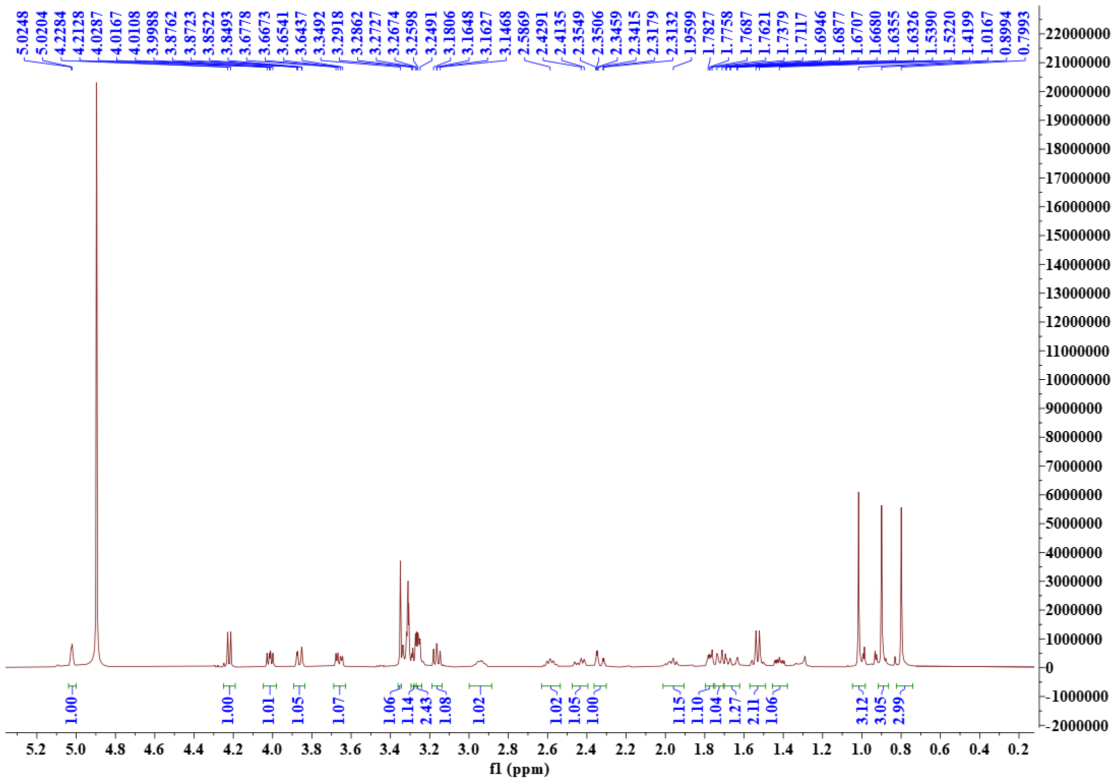

Fig. S7 ^{1}H NMR (500 MHz) spectrum of compound **1** in CD_3OD.

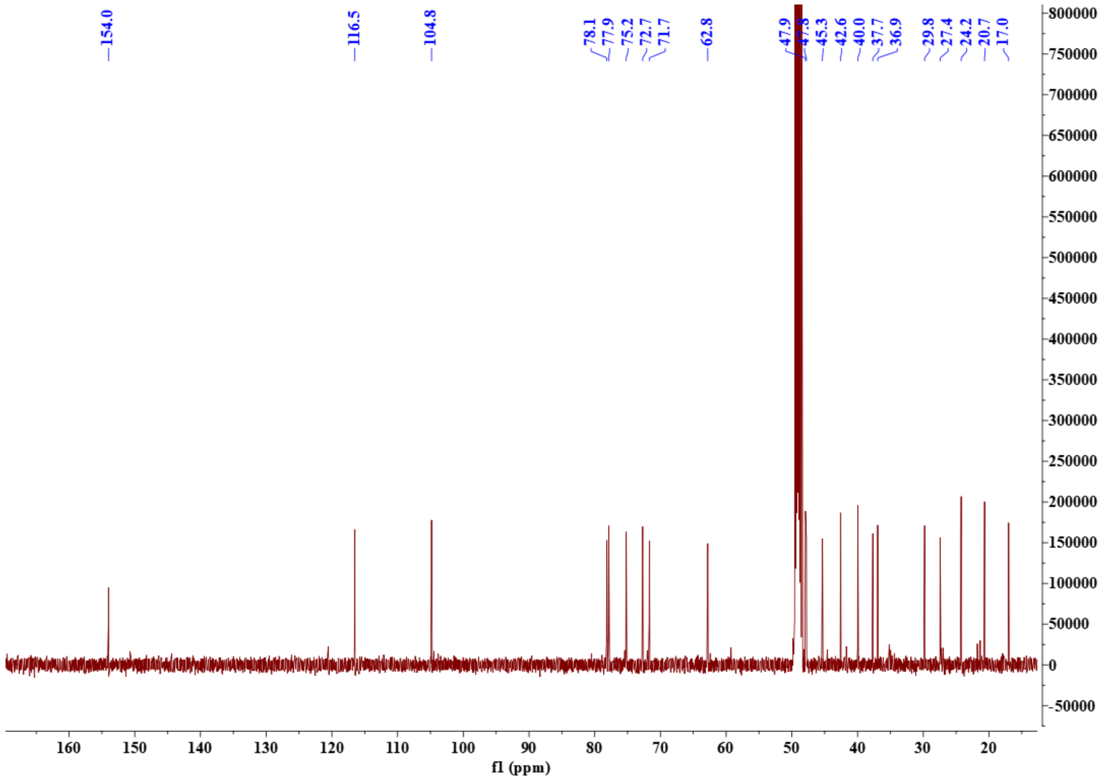

Fig. S8 ^{13}C NMR (125 MHz) spectrum of compound **1** in CD_3OD.

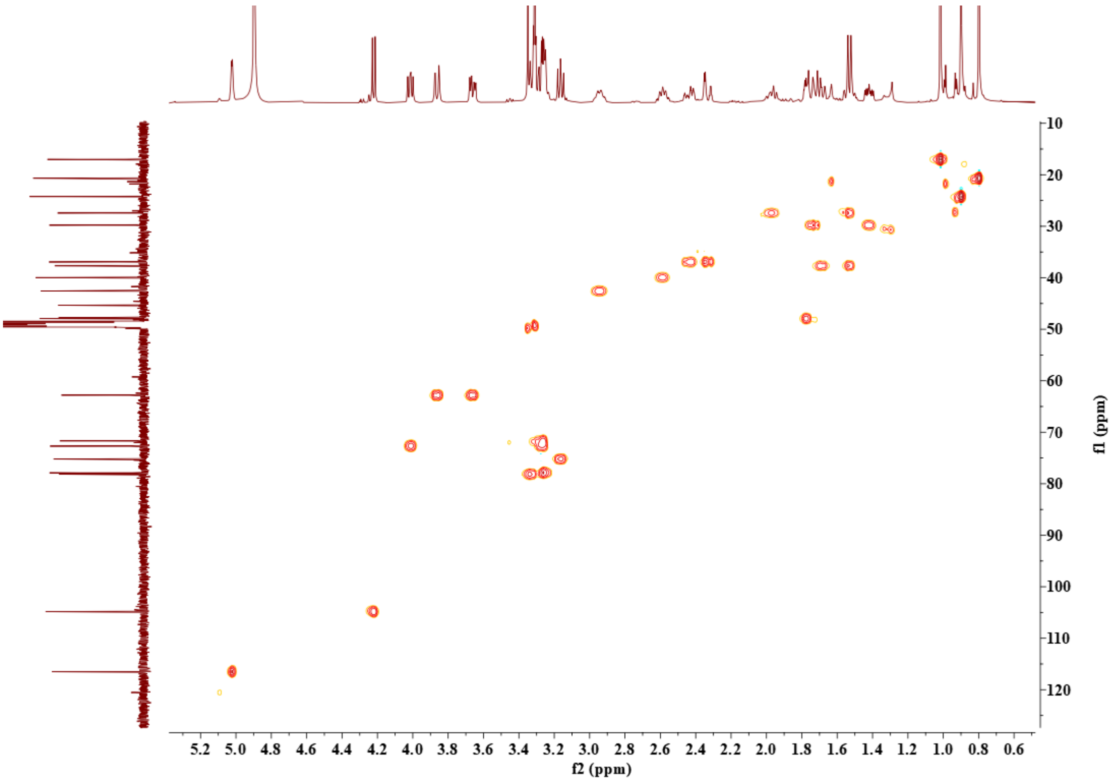

Fig. S9 HSQC spectrum of compound **1** in CD_3OD.

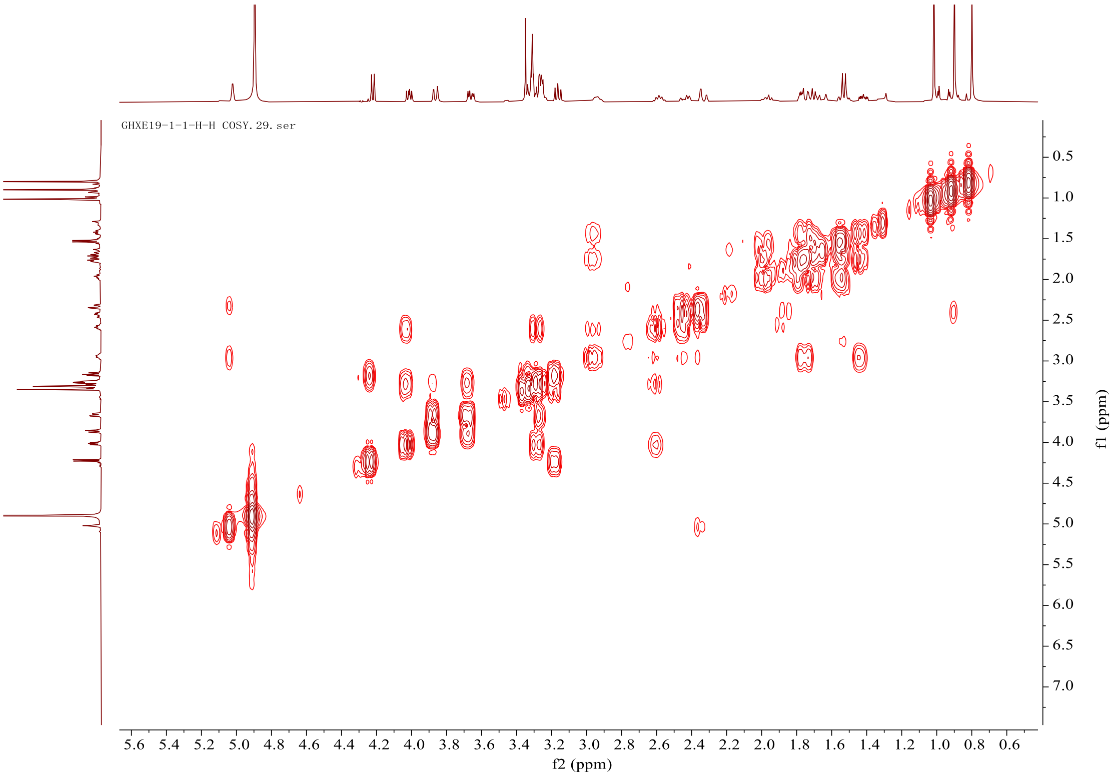

Fig. S10 ^1H-^1H COSYspectrum of compound **1** in CD_3OD.

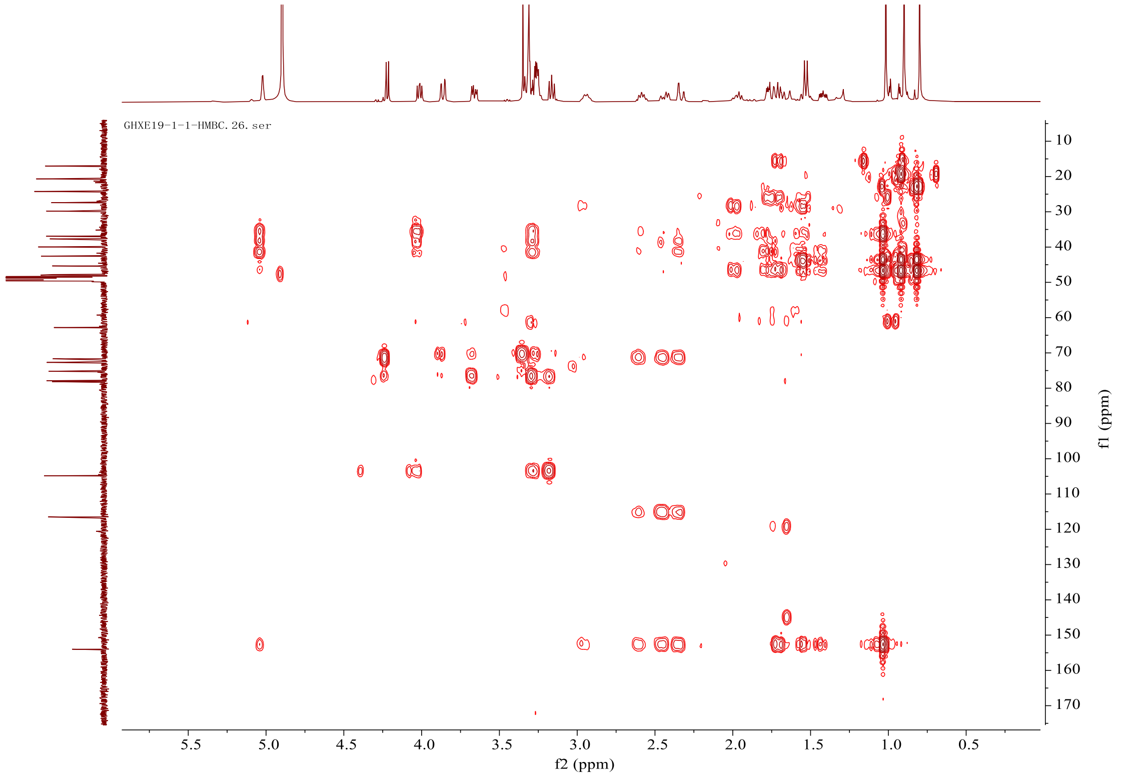

Fig. S11 HMBC spectrum of compound **1** in CD_3OD.

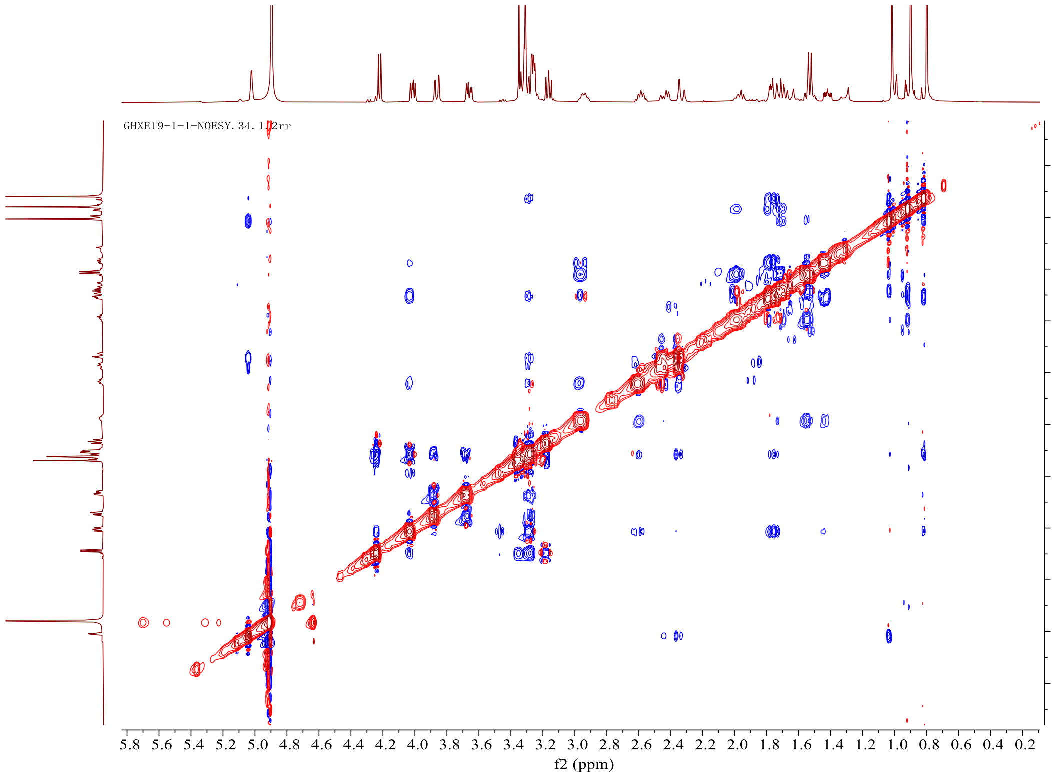

Fig. S12 NOESY spectrum of compound **1** in CD_3OD.

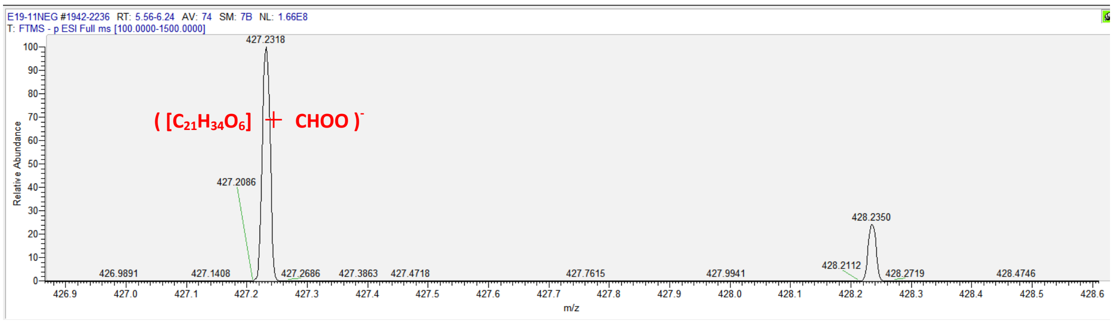

m/z	*Calc m/z*	Delta mmu(ppm)	z	Abund	Formula	Ion
427.2318	427.2326	-0.814	1	166000000	$C_{22}H_{35}O_8$	$(M+CHOO)^-$

Fig. S13 HRESIMS of compound **1**.

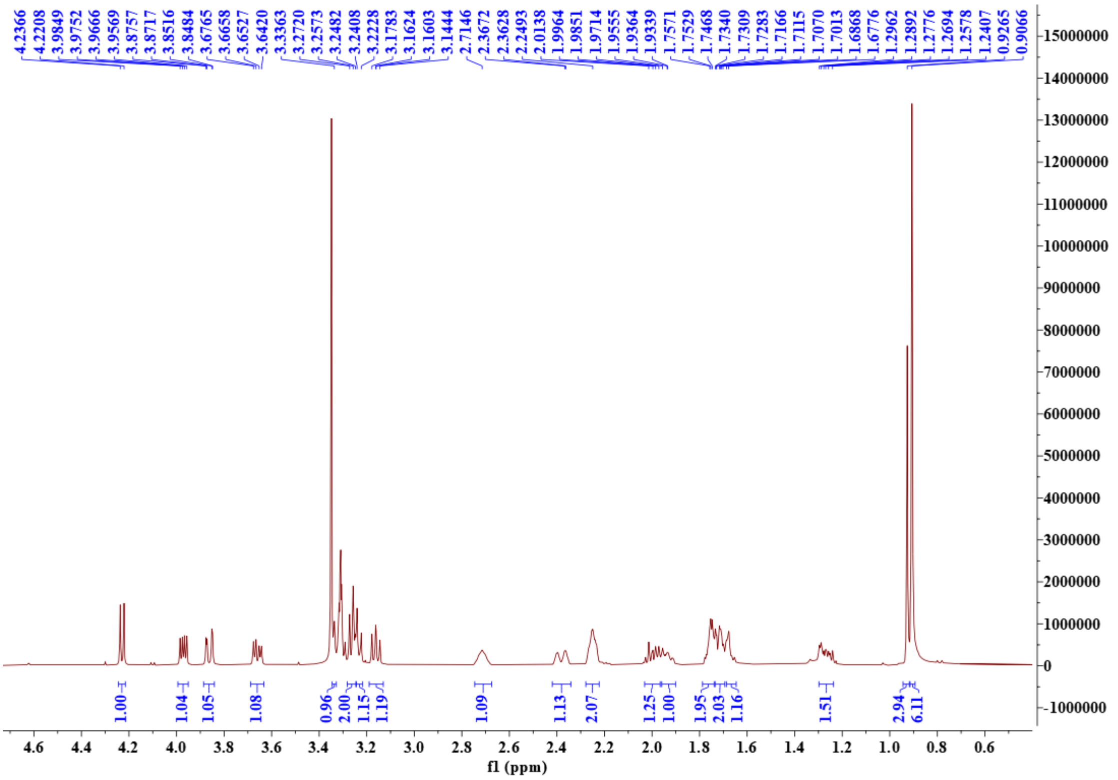

Fig. S14 ^{1}H NMR (500 MHz) spectrum of compound **2** in CD_3OD.

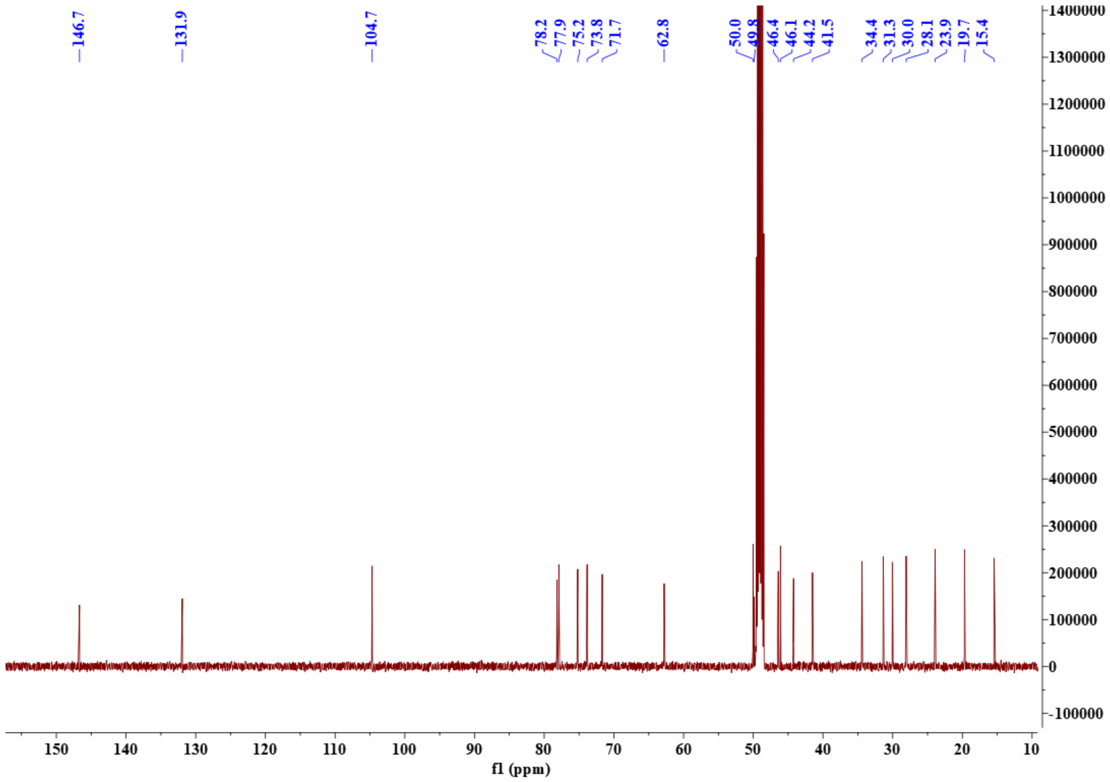

Fig. S15 ^{13}C NMR (125 MHz) spectrum of compound **2** in CD_3OD.

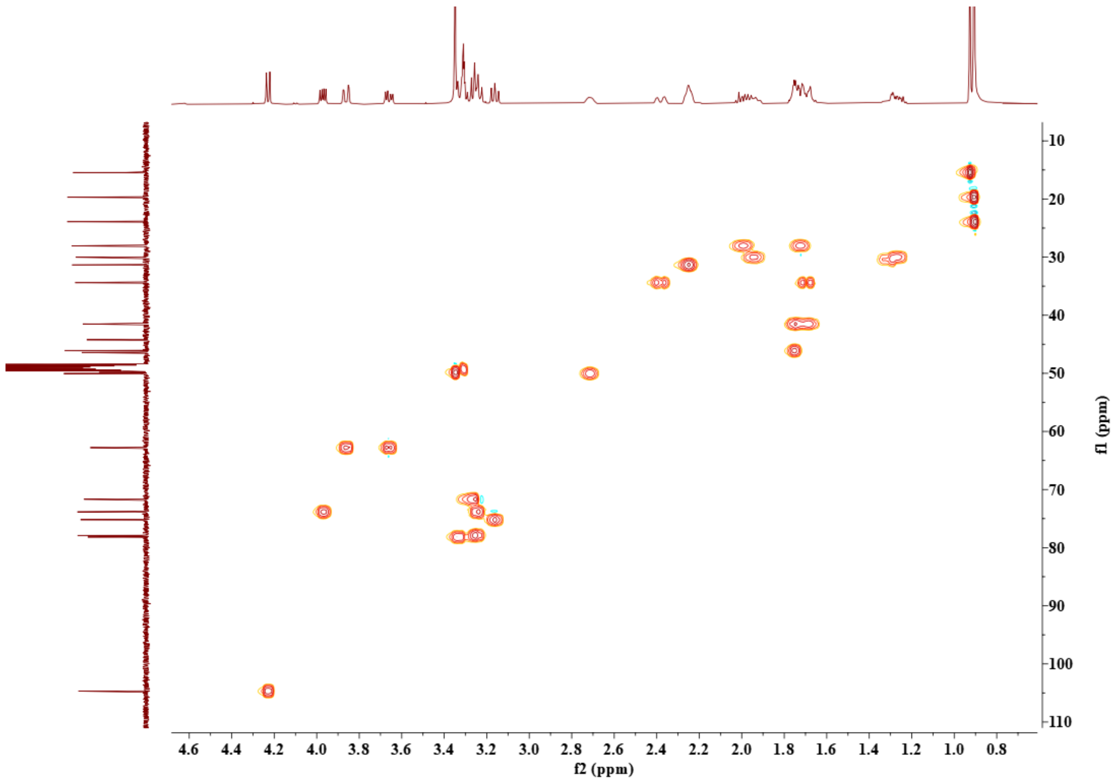

Fig. S16 HSQC spectrum of compound **2** in CD_3OD.

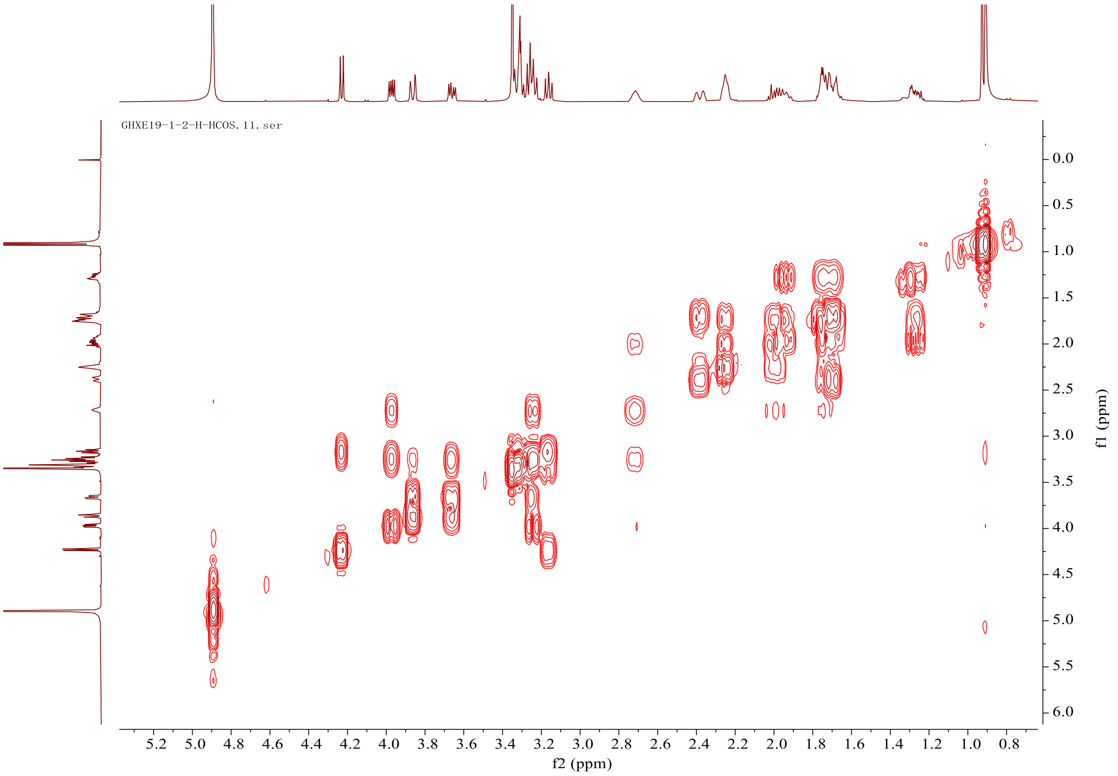

Fig. S17 ^{1}H-^{1}H COSYspectrum of compound **2** in CD_3OD.

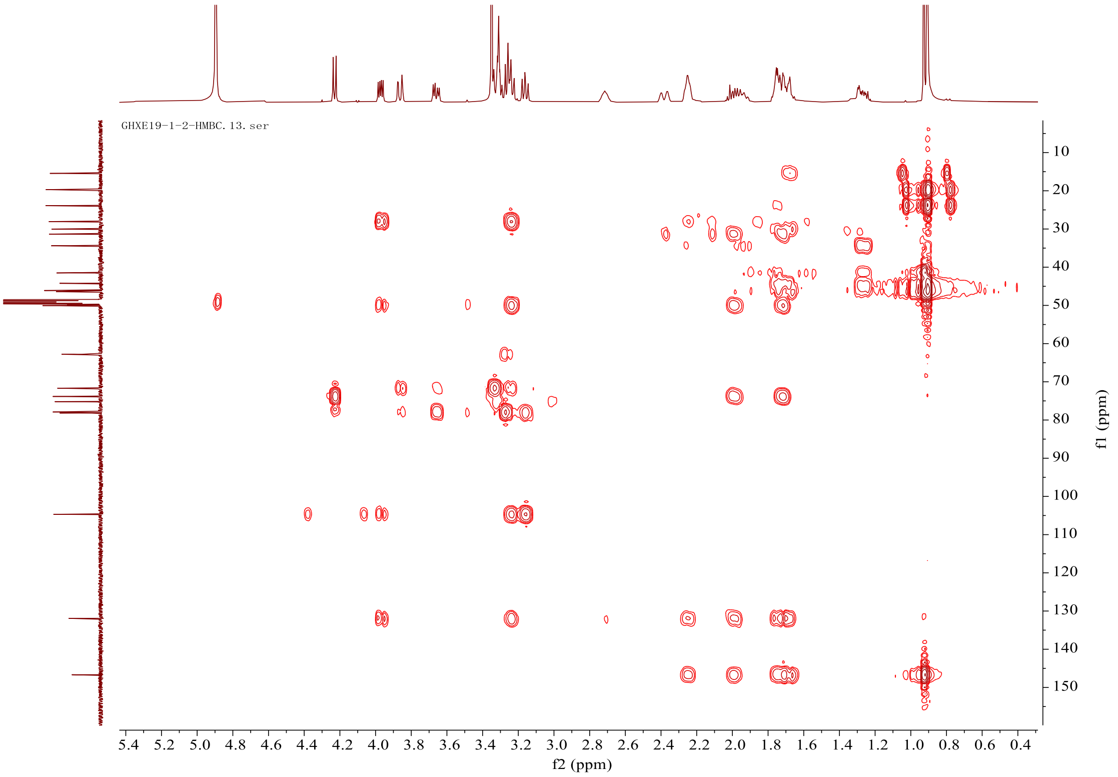

Fig. S18 HMBC spectrum of compound **2** in CD_3OD.

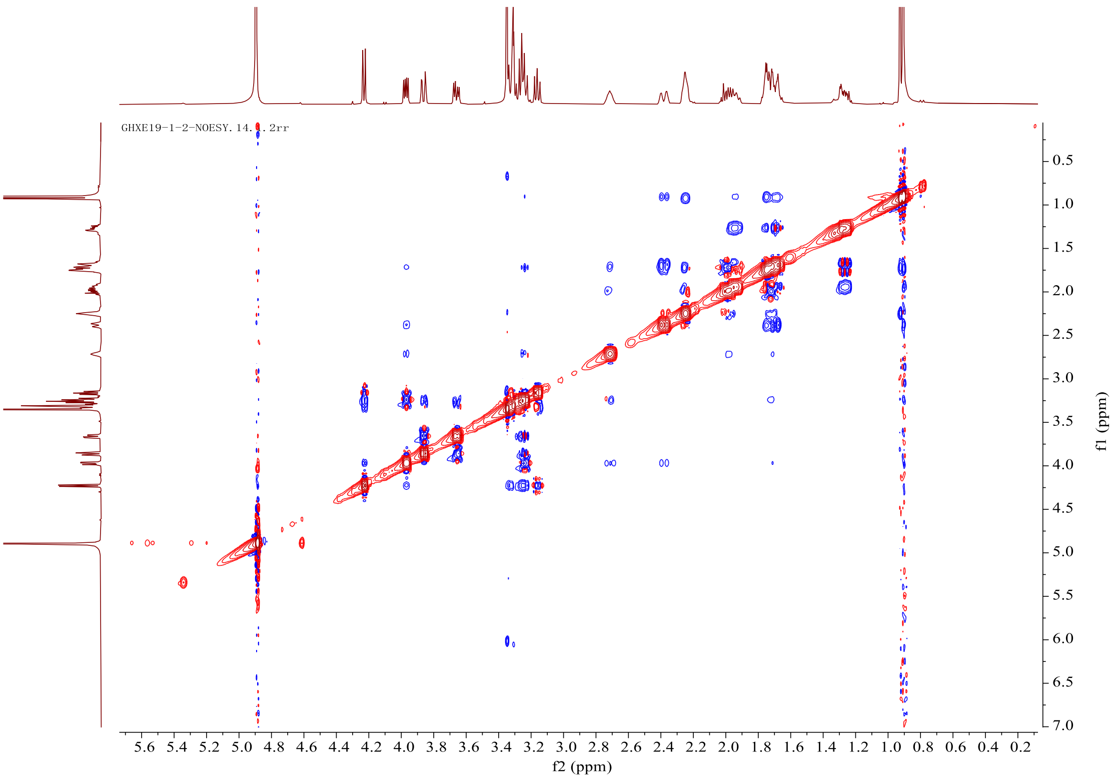

Fig. S19 NOESY spectrum of compound **2** in CD_3OD.

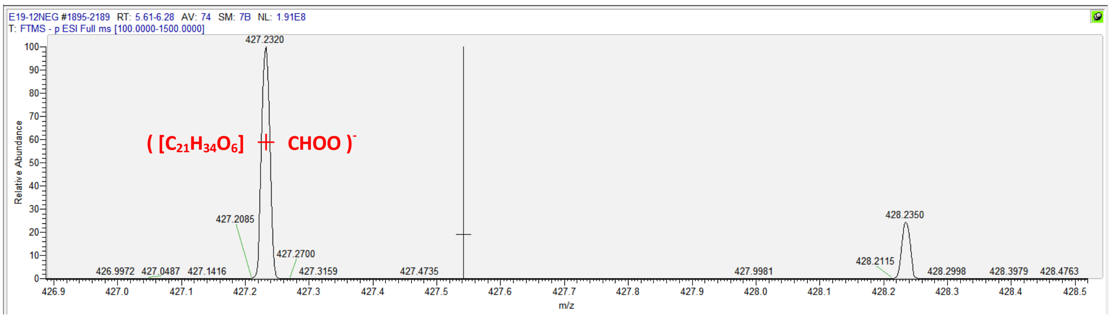

m/z	*Calc m/z*	Delta mmu(ppm)	z	Abund	Formula	Ion
427.2320	427.2326	-0.644	1	190000000	$C_{22}H_{35}O_8$	$(M+CHOO)^-$

Fig. S20 HRESIMS of compound **2**.

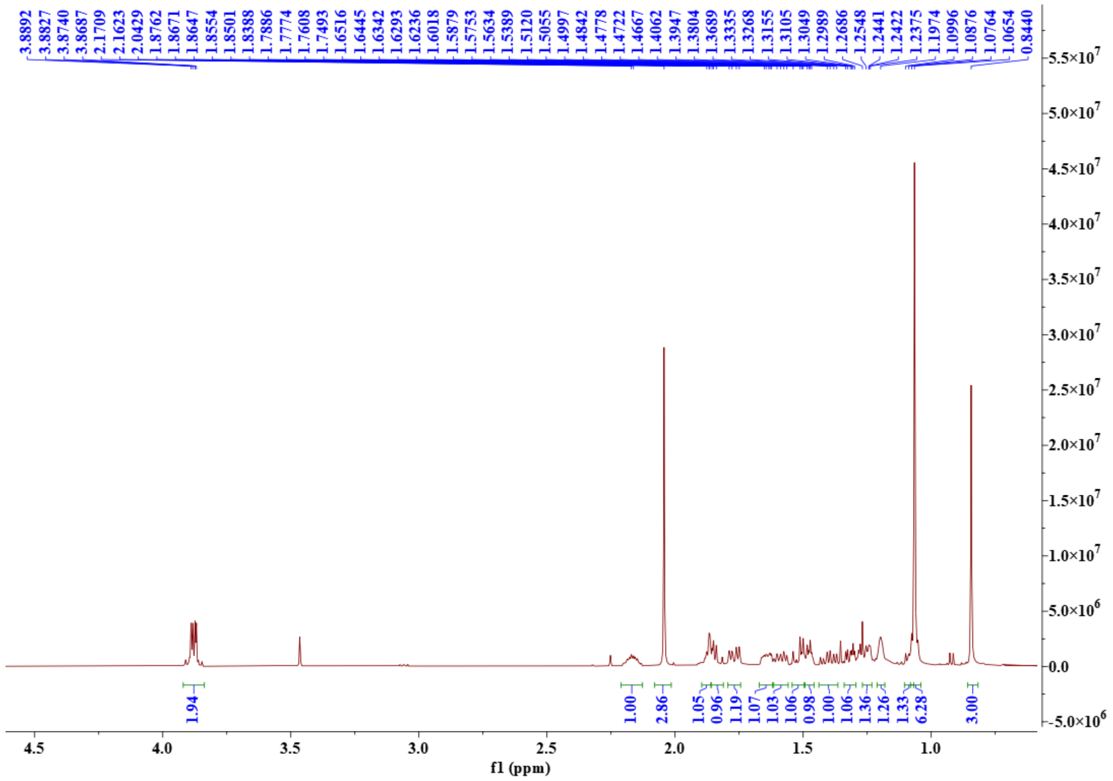

Fig. S21 ^{1}H NMR (500 MHz) spectrum of compound **3** in $CDCl_3$.

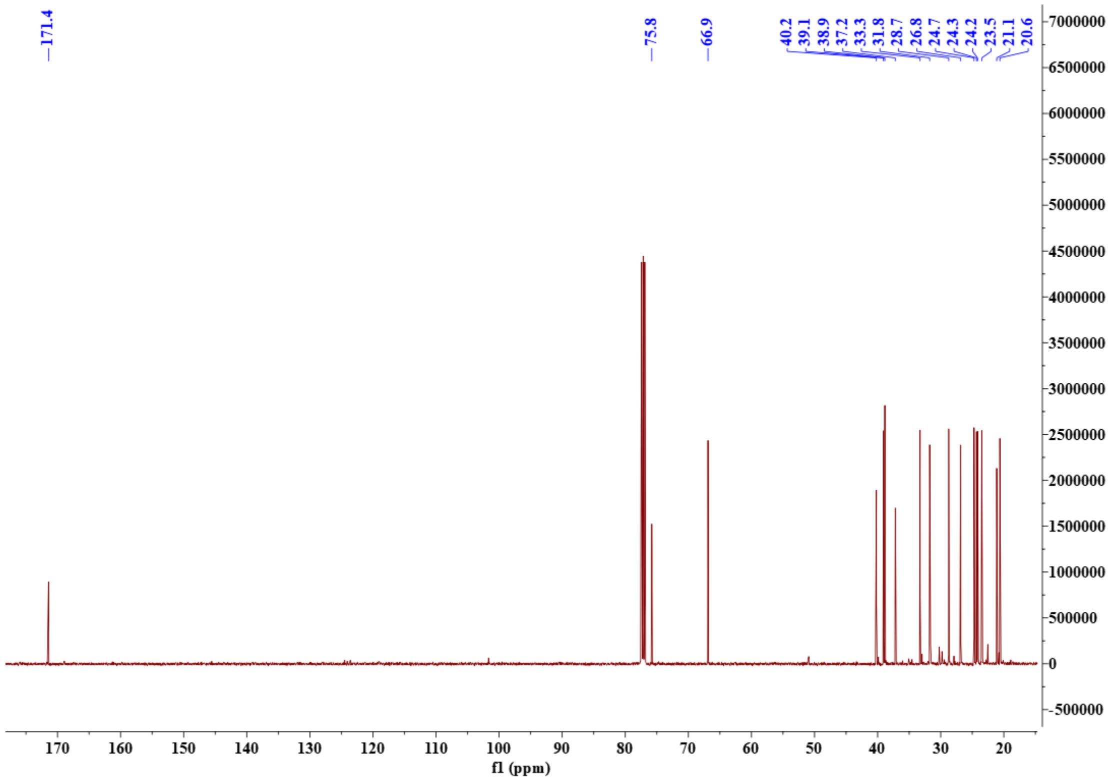

Fig. S22 ^{13}C NMR (125 MHz) spectrum of compound **3** in $CDCl_3$.

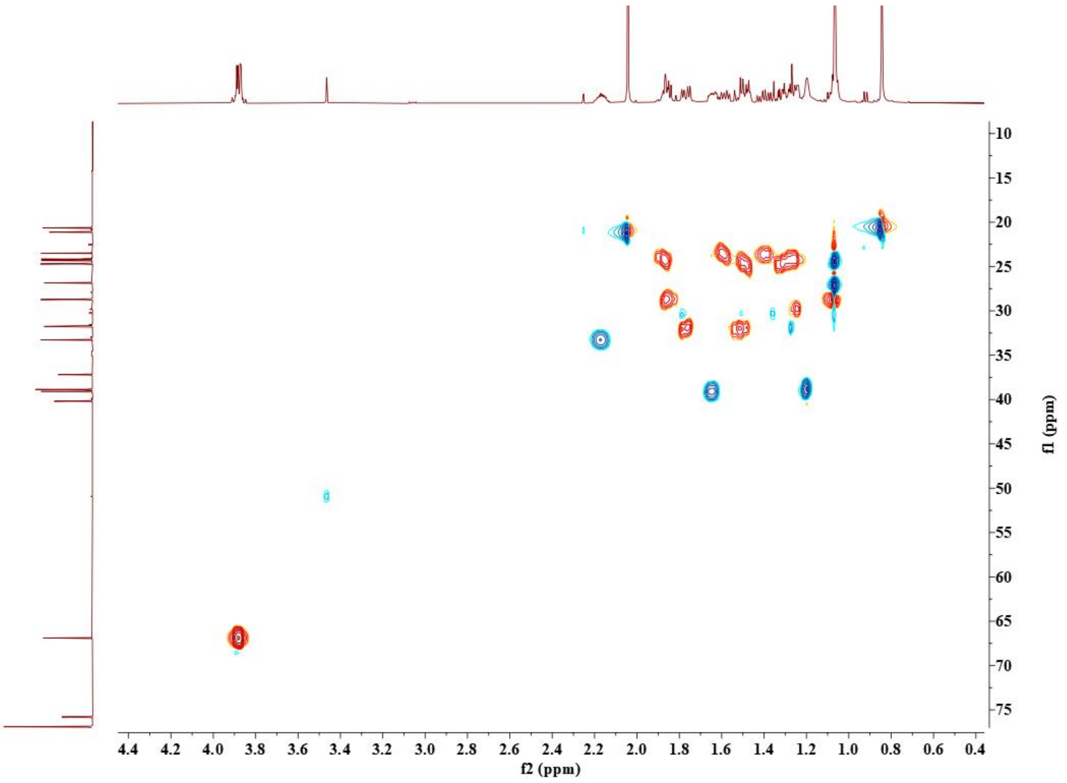

Fig. S23 HSQC spe ctrum of compound **3** in $CDCl_3$.

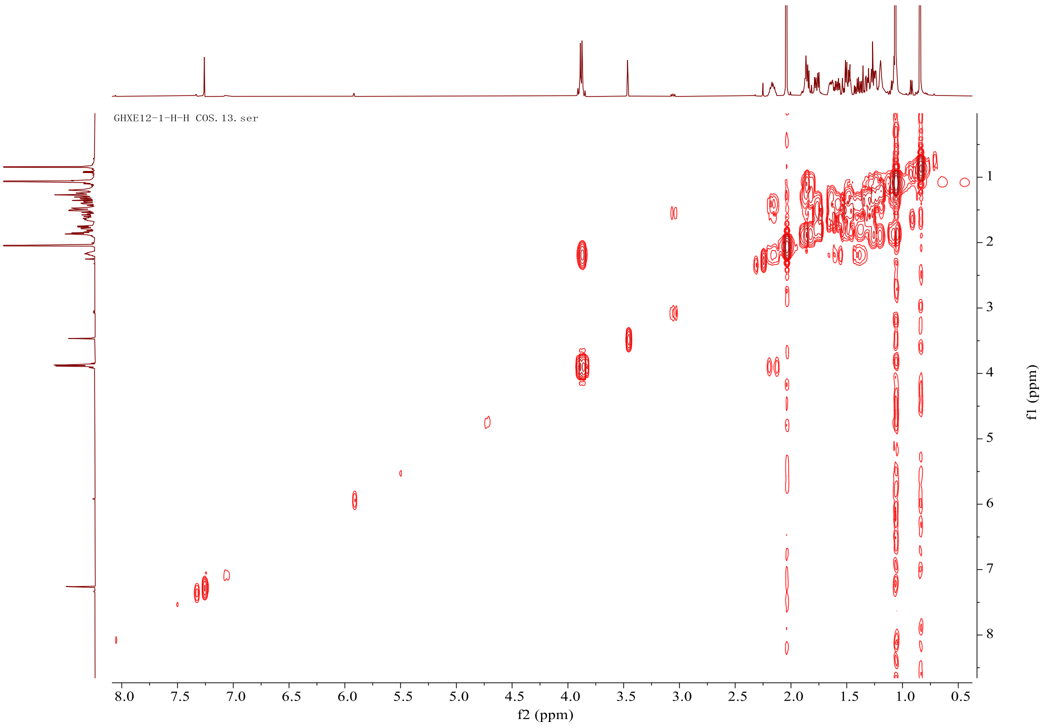

Fig. S24 ^1H-^1H COSYspectrum of compound **3** in $CDCl_3$.

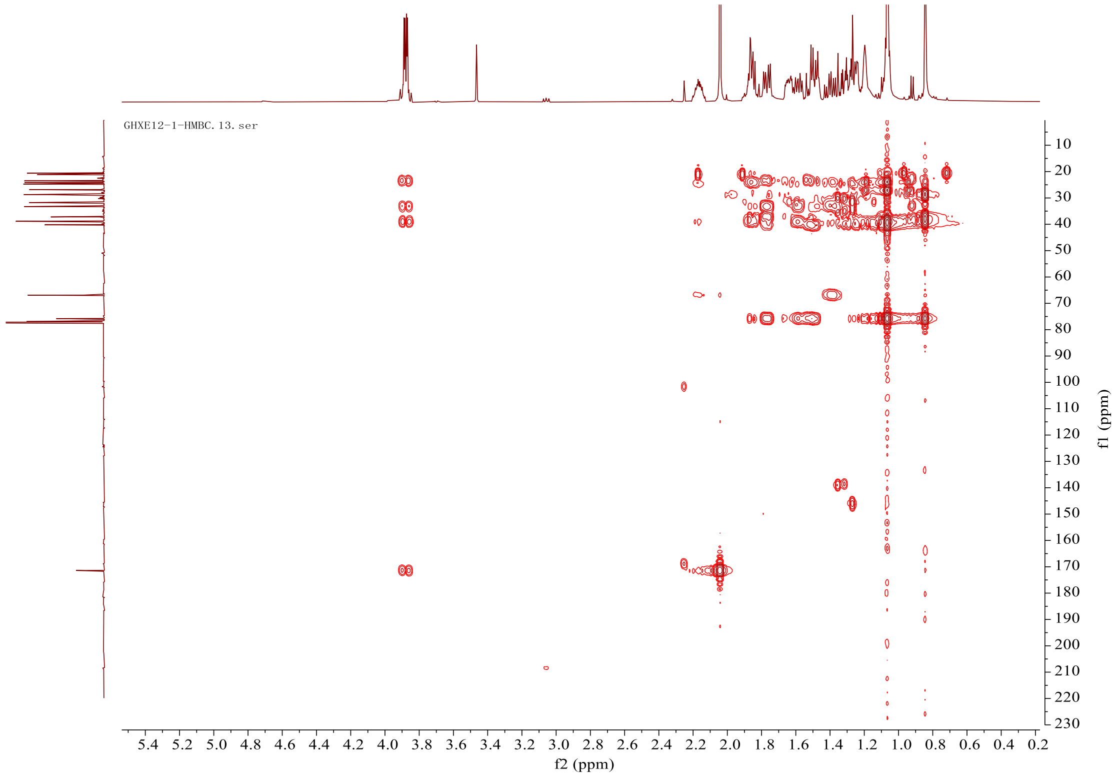

Fig. S25 HMBC spectrum of compound **3** in $CDCl_3$.

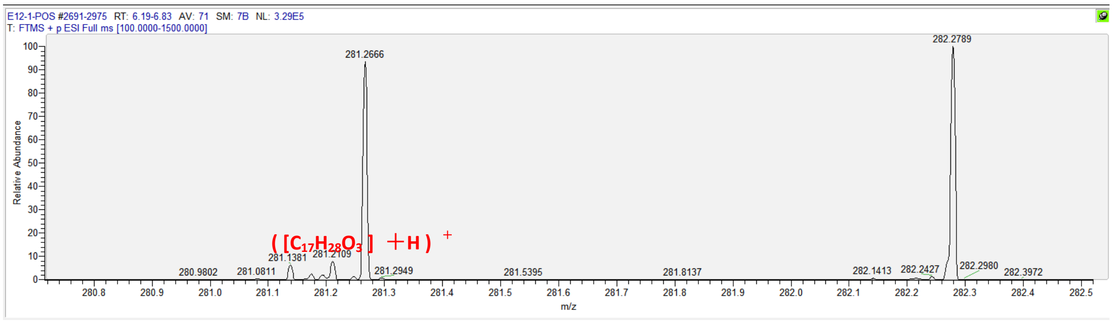

m/z	*Calc m/z*	Delta mmu(ppm)	z	Abund	Formula	Ion
281.2109	281.2111	-0.0141	1	329000	$C_{17}H_{29}O3$	$(M+H)^+$

Fig. S26 HRESIMS of compound **3**.

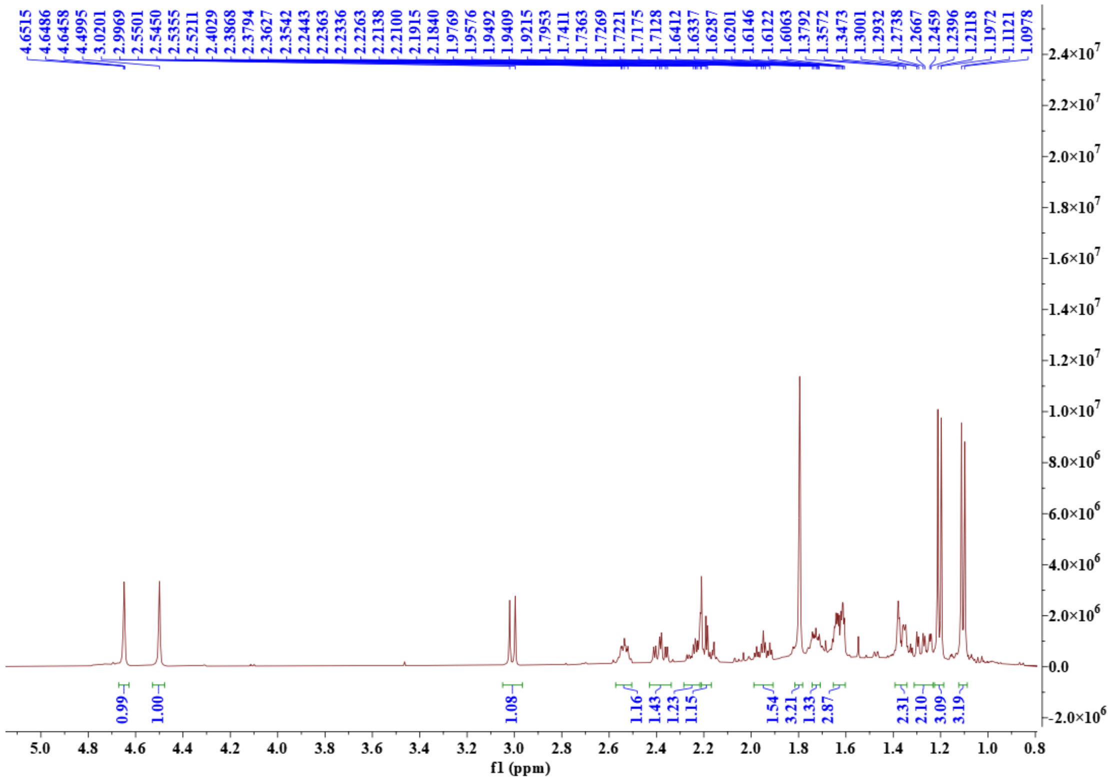

Fig. S27 ^{1}H NMR (500 MHz) spectrum of compound **4** in $CDCl_3$.

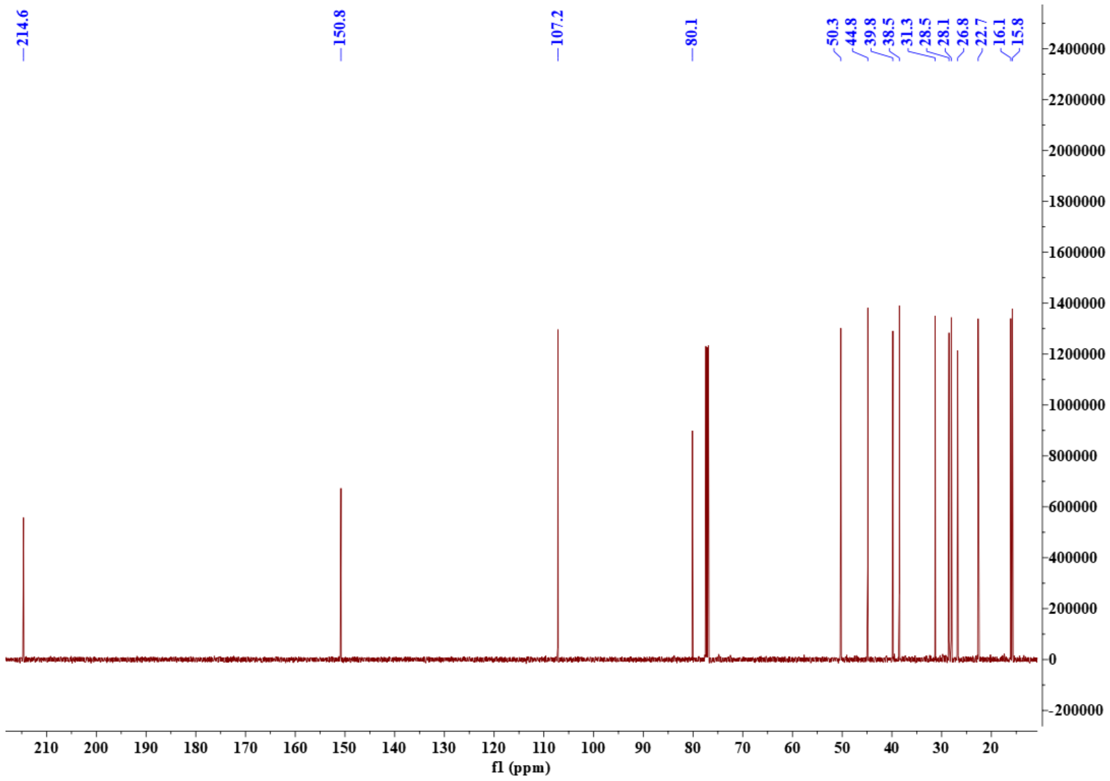

Fig. S28 ^{13}C NMR (125 MHz) spectrum of compound **4** in $CDCl_3$.

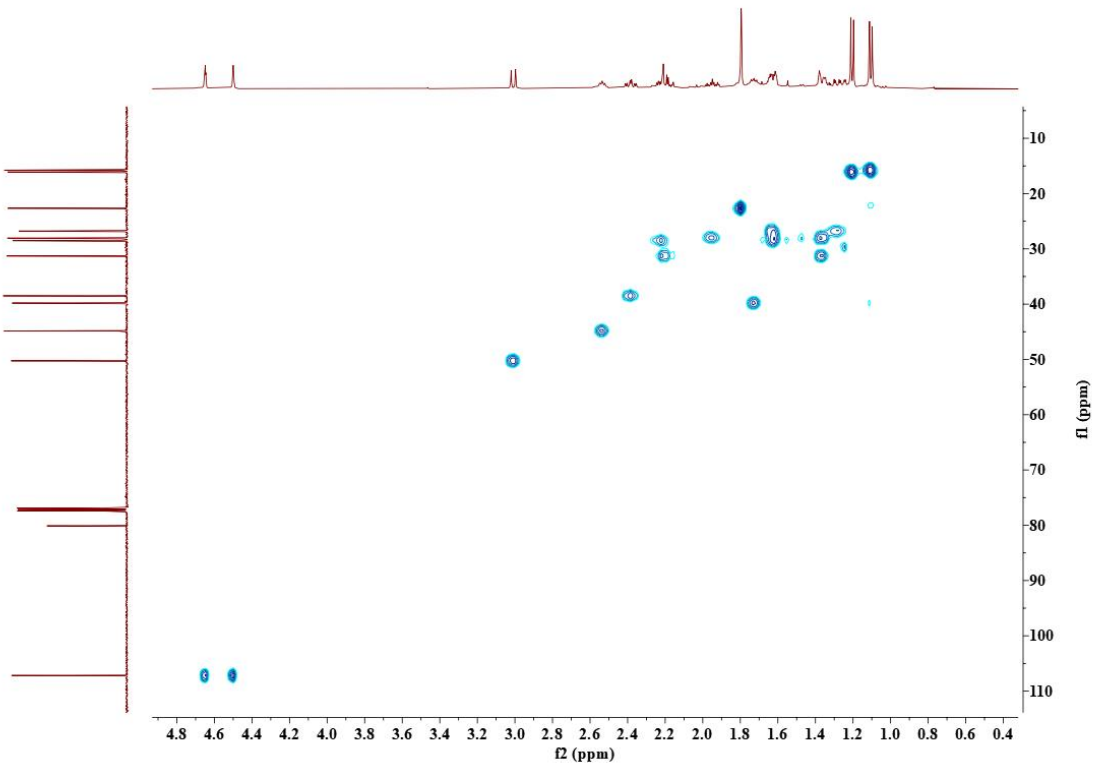

Fig. S29 HSQC spectrum of compound **4** in $CDCl_3$.

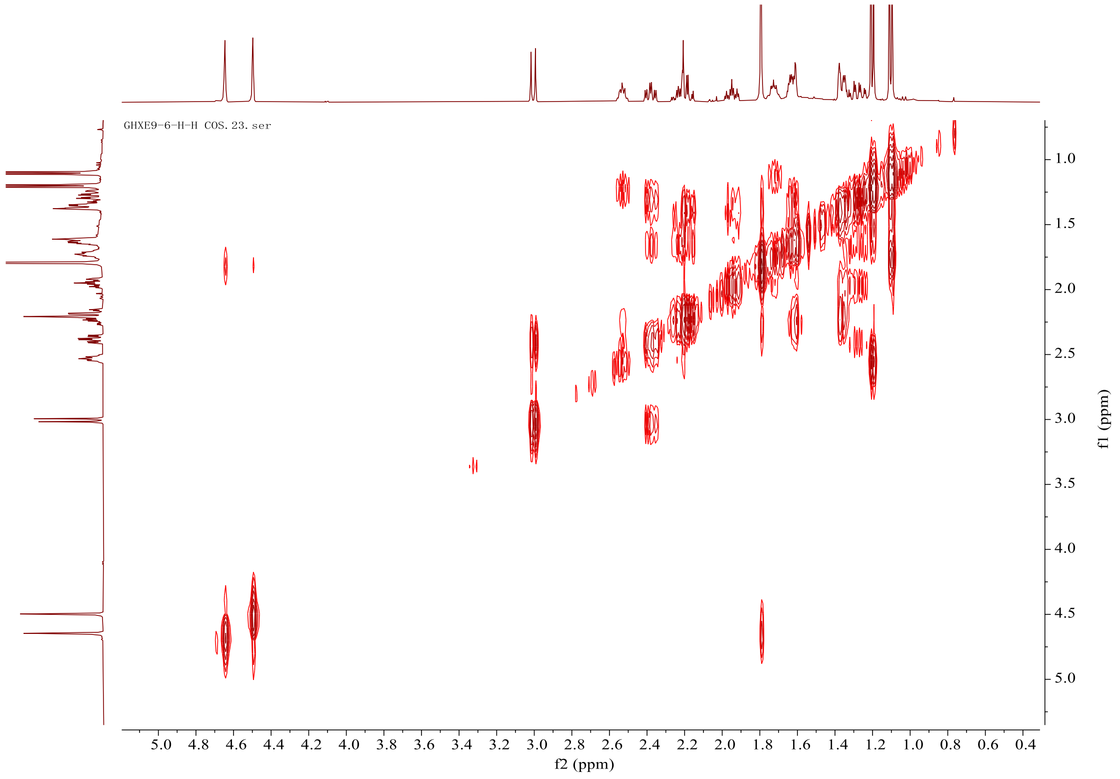

Fig. S30 ^{1}H-^{1}H COSYspectrum of compound **4** in $CDCl_3$.

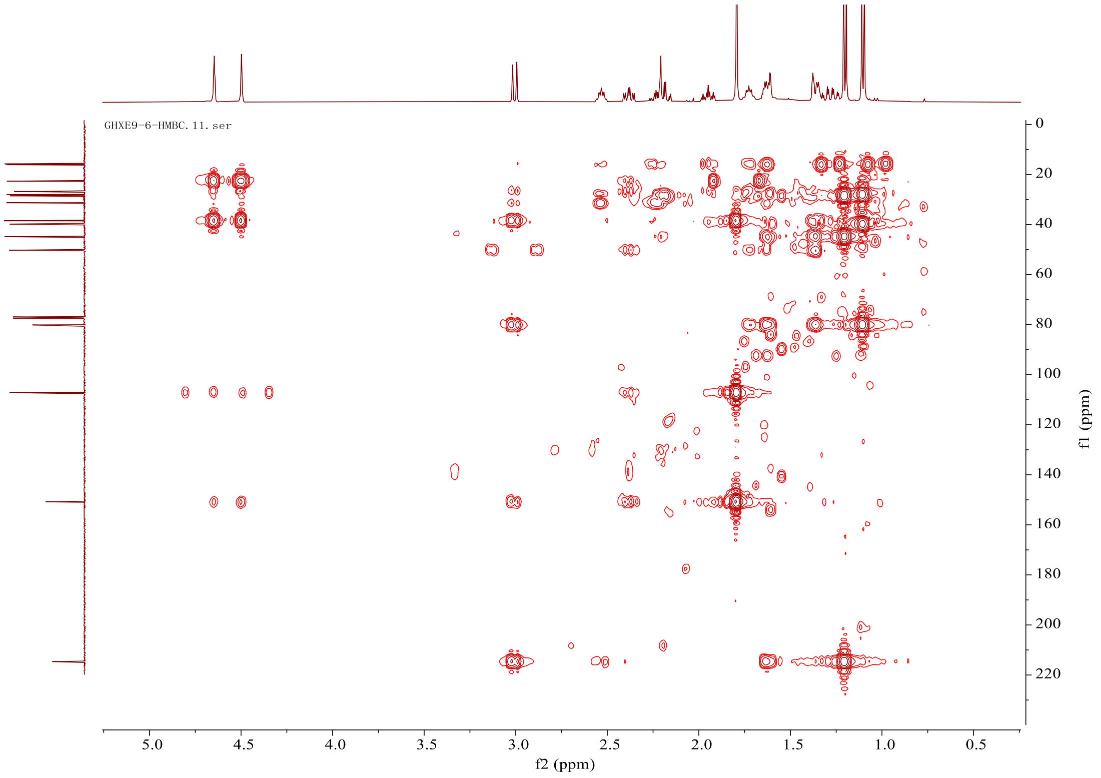

Fig. S31 HMBC spectrum of compound **4** in $CDCl_3$.

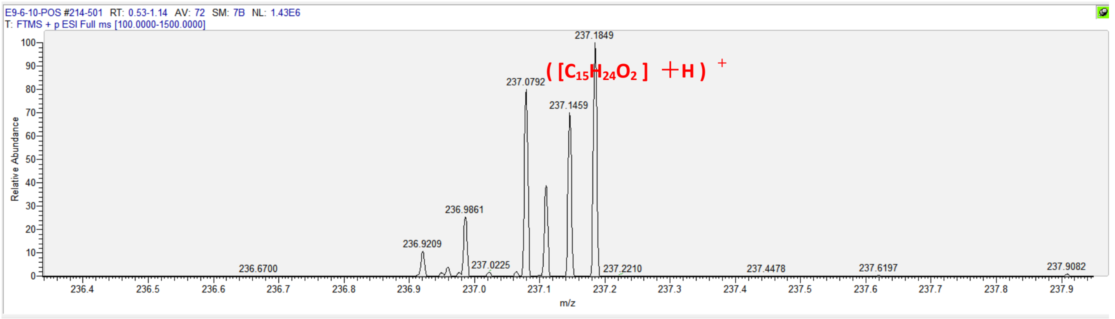

m/z	*Calc m/z*	Delta mmu(ppm)	z	Abund	Formula	Ion
237.1849	237.1849	-0.037	1	675000	$C_{15}H_{25}O_2$	$(M+H)^+$

Fig. S32 HRESIMS of compound **4**.
